# Genome-Wide Differentiation, Inbreeding, and Candidate Selection Loci in Local Vietnamese Pig Breeds

**DOI:** 10.3390/ijms27177897

**Published:** 2026-09-04

**Authors:** Van Thanh Nguyen, Nguyen Van Ba, Duy Ngoc Do

**Affiliations:** 1 Faculty of Veterinary Medicine, Viet Nam National University of Agriculture, Hanoi 100000, Vietnam; 2 Key Laboratory of Animal Cell Technology, Vietnam Institute of Animal and Veterinary Sciences, Thuongcat, Hanoi 100000, Vietnam; nguyenba81@yahoo.com; 3 Institute of Research and Development, Duy Tan University, Da Nang 550000, Vietnam; 4 School of Medicine and Pharmacy, Duy Tan University, Da Nang 550000, Vietnam; 5 Department of Animal Science and Aquaculture, Dalhousie University, Truro, NS B2N 5E3, Canada

**Keywords:** selection signatures, FST, XP-EHH, ROH, PCA, Vietnamese pigs, Sus scrofa, local breeds, candidate selection signatures, putative tropical adaptation, exploratory population genomics, coat color, body size

## Abstract

Vietnam harbors exceptional genetic diversity among at least 26 indigenous pig breeds. We analyzed genome-wide single-nucleotide polymorphism (SNP) data from 90 animals representing 15 local Vietnamese breeds and six Landrace pigs using principal component analysis, the windowed fixation index (FST), cross-population extended haplotype homozygosity (XP-EHH), within-population integrated haplotype score (iHS), and runs of homozygosity (ROHs). The population structure was consistent with a north–south differentiation axis, and Ba Xuyen showed elevated heterozygosity, providing suggestive evidence of a European genetic contribution; the f3 statistic was positive (f3 = +0.015), and formal evidence of admixture requires a significantly negative f3, so this criterion was not met. Integration of FST and XP-EHH identified *GPC5*, *E2F6*, *NOS1*, and TLR4 as top Northern candidate loci and *CRYM*/*ZP2* as the leading Central candidate locus, and these windows were recovered at both the 90th and 95th percentile thresholds, indicating analytical robustness rather than independent biological validation. iHS was elevated at *E2F6* in Northern breeds (|iHS| = 3.04) and at *NOS1* across all regional groups (|iHS| = 2.66–3.36). Breed-level phenotypic XP-EHH, based on published breed descriptions and coat color rather than individual body-composition measurements, identified *GALNT2* as a candidate shared across breed groups; *HCAR1* and *ATG10* as candidates specific to the extreme-fat/prolific breed group; and *EFNA5* and *HIPK2* as candidates specific to the medium-bodied breed group. ROHs identified Soc, Co, and Hung as breeds warranting particular attention in conservation planning due to elevated autozygosity. Because each breed was represented by only six individuals, and because no individual-level phenotypic measurements were available, all findings are reported as exploratory population-genomic signals requiring replication in larger cohorts. Overall, we describe genomic differentiation and candidate selection signatures among local Vietnamese pig breeds and provide a foundation for further genomic studies of these breeds.

## 1. Introduction

Vietnam is recognized as a secondary center of pig diversification in Southeast Asia, with at least 26 indigenous breeds distributed across the country’s varied geographic and ecological zones [1]. These breeds have evolved over millennia under diverse selection pressures from the cool montane highlands of the north to the tropical river deltas of the south, resulting in substantial phenotypic diversity. They vary markedly in body size (small to large), coat color (black, spotted, white-patched), backline morphology (slim/drooping to swayback), litter size (4–16 piglets), and conservation risk status. Additionally, these breeds show distinct production orientations. Highland breeds such as Ban, Muong Khuong, and Lung Pu are typically small-bodied, lean, and valued for meat quality and foraging ability. In contrast, breeds such as Mong Cai and Ha Lang show larger body size, higher fat deposition, and exceptionally high litter sizes (10–16 for Mong Cai). Therefore, they might have distinct selection histories focused on reproductive output and fatness. Understanding the genomic basis of this within-Vietnamese phenotypic diversification, alongside the divergence from European commercial breeds such as Landrace, is therefore relevant to both conservation management and sustainable production improvement [2].

Genome-wide single-nucleotide polymorphism (SNP) data provide a powerful means to detect signatures of selection in local pig populations, revealing the genomic regions and genes that have been targeted by both natural and artificial selection during breed formation and adaptation [3]. In pigs, SNP-based scans have uncovered strong selection around loci associated with key production and morphological traits [4,5]. The NR6A1 region on chromosome 1 has been repeatedly reported as a major selective sweep linked to increased vertebral number and body length in domestic pigs [6]. Fontanesi and Russo (2013) confirmed that variants at the MC1R and KIT loci determine coat color phenotypes in Mediterranean pigs [7]. Zhu et al. [8] identified *TBX19*, *AHR*, *MSTN*, *P2RY1*, *MARCH1*, *OR11L1*, and *OR14A16* as potential contributors to the adaptation to capture life and the response to artificial selection of human-desired traits in Chinese domestic pigs. Applying similar SNP-based selection-signature approaches to local Vietnamese pig populations can help identify genomic regions underlying locally important traits, such as disease resistance, heat tolerance, and meat quality, thereby informing both conservation strategies and targeted genetic improvement programs. Disease resistance, heat tolerance, and meat quality were not directly measured in the present dataset; rather, they provide the biological motivation for identifying genomic regions that may underlie locally important traits.

The pigs were domesticated in several places, including the Near East, China, and Southeast Asia [9,10]. Artificial selection has produced a wide variety of distinct phenotypes, including fast growth, high reproductive capacity, and altered external appearance. Although these breeds are important for conservation, major studies in Vietnamese pigs have been based on microsatellites or mtDNA [11,12,13]. SNP arrays have been used only for population structure analyses [14]. However, little is known about selection signatures, autozygosity levels, or the genomic basis of within-Vietnamese phenotypic differentiation in these breeds. Here, we used a publicly available dataset of 90 local Vietnamese pigs from 15 breeds and 6 Landrace pigs genotyped on the Illumina PorcineSNP60v2 BeadChip to conduct the first comprehensive selection signature analysis. This study therefore aimed to (i) characterize the population structure using principal component analysis (PCA), which resolves major axes of genetic variation among individuals; (ii) quantify regional genetic differentiation using pairwise FST, a measure of allele-frequency divergence between populations; (iii) identify selection signals relative to Landrace using windowed FST and XP-EHH; (iv) detect within-Vietnamese selection signals associated with phenotypic diversity using within-group FST, corroborated by within-population integrated haplotype score (iHS); and (v) assess autozygosity using runs of homozygosity (ROHs) to identify breeds warranting particular attention in conservation planning. Because each breed is represented by only six individuals, because phenotypic groups were assigned from published breed descriptions rather than from individual measurements, and because no functional validation was performed, we emphasize at the outset that the results presented here are exploratory population-genomic signals and candidate loci rather than demonstrated biological mechanisms or validated adaptive phenotypes.

## 2. Results

### 2.1. Dataset Composition and Genotype Quality

After all quality control filters, 48,896 autosomal SNPs were retained across 96 individuals (Table 1). No individuals were removed. The per-breed mean genotyping rate ranged from 0.992 (Soc) to 0.998 (Landrace; Supplementary Table S1). Mean observed heterozygosity ranged from 0.164 (Soc) to 0.366 (Lung) among local Vietnamese breeds; Ba_Xuyen showed markedly higher heterozygosity (0.388), providing suggestive evidence of a European genetic contribution. No related pairs (pi-hat > 0.25) or duplicate individuals were detected. PCA was performed on 9901LD-pruned SNPs; all 48,896 SNPs were used for FST, XP-EHH, and ROH analyses. Each of the 16 breeds (15 local Vietnamese breeds and Landrace) was represented by six individuals; geographic region and phenotypic group assignments are given in Section 4.1 and Section 4.6, respectively, and the full per-breed genotyping rate and heterozygosity are provided in Supplementary Table S1. It is important to note that each breed was represented by only six individuals, whereas the pooled regional and phenotype-related groups comprised larger numbers of animals; all population-genomic estimates reported below are therefore accompanied by substantial sampling uncertainty and should be regarded as exploratory.

### 2.2. Population Structure

**Figure 1:**
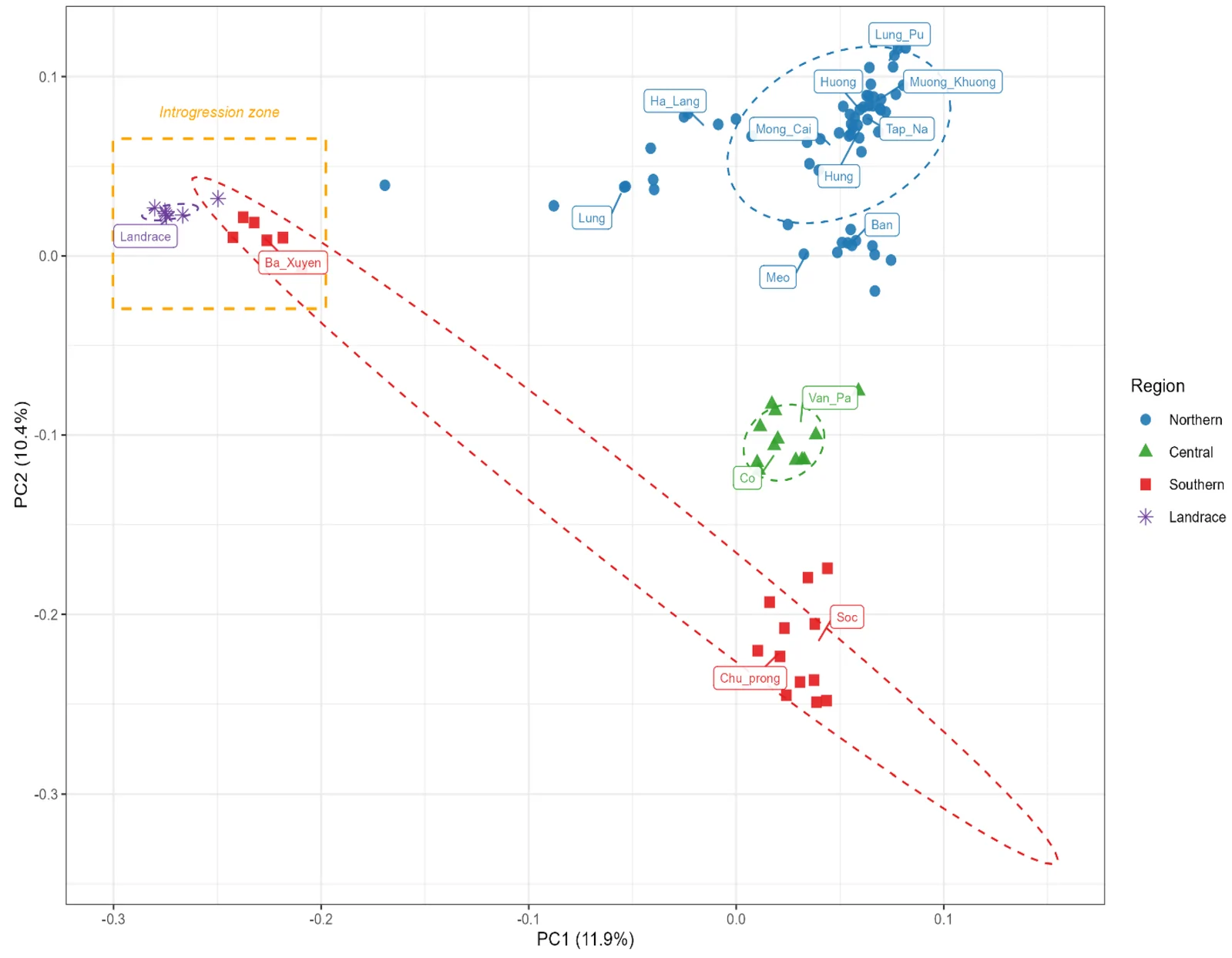
PCA of 15 Vietnamese pig breeds and Landrace (9901 LD-pruned SNPs). PC1 (11.9%) separates Asian from European lineages; PC2 (10.4%) reflects north–south geographic structure. Dashed ellipses = 75% confidence regions. Orange box highlights the region of principal component space in which Ba Xuyen clusters towards Landrace; this pattern provides suggestive evidence of a European genetic contribution rather than formally demonstrated introgression.

PCA of 9,901 LD-pruned SNPs showed structure consistent with geographic origin (Figure 1). PC1 (11.9%) separated Landrace from all Vietnamese breeds; PC2 (10.4%) captured a north–south gradient within Vietnamese breeds (Figure S1). Ba Xuyen clustered intermediately between Landrace and Southern Vietnamese breeds on PC1, a pattern providing suggestive evidence of a European genetic contribution. To formally test this, we computed f3(Ba_Xuyen; Landrace, Vietnamese_reference) = +0.015 (SE = 0.0004, Z = +39.9). The positive f3 does not meet the formal criterion for admixture, which requires a significantly negative f3 (f3 < 0 with Z < −3), and the present data therefore provide no formal f3 evidence of admixture in Ba Xuyen. However, it is not clear how strongly allele-frequency variance at *n* = 6 inflates f3 estimates in this dataset, but we speculate that the small sample size might have influenced our results. In this study, the convergent PCA, FST, and heterozygosity patterns are therefore described as suggestive evidence of a European genetic contribution to Ba Xuyen rather than as confirmed European admixture, and formal admixture testing in larger cohorts is required. Soc occupied an extreme position on PC2 and showed the highest genome-wide inbreeding coefficient (FROH) among Southern breeds (Section 2.5), suggesting that its distinct genomic profile reflects both regional differentiation and demographic isolation.

### 2.3. Genetic Differentiation Between Regional Groups and Landrace

**Figure 2:**
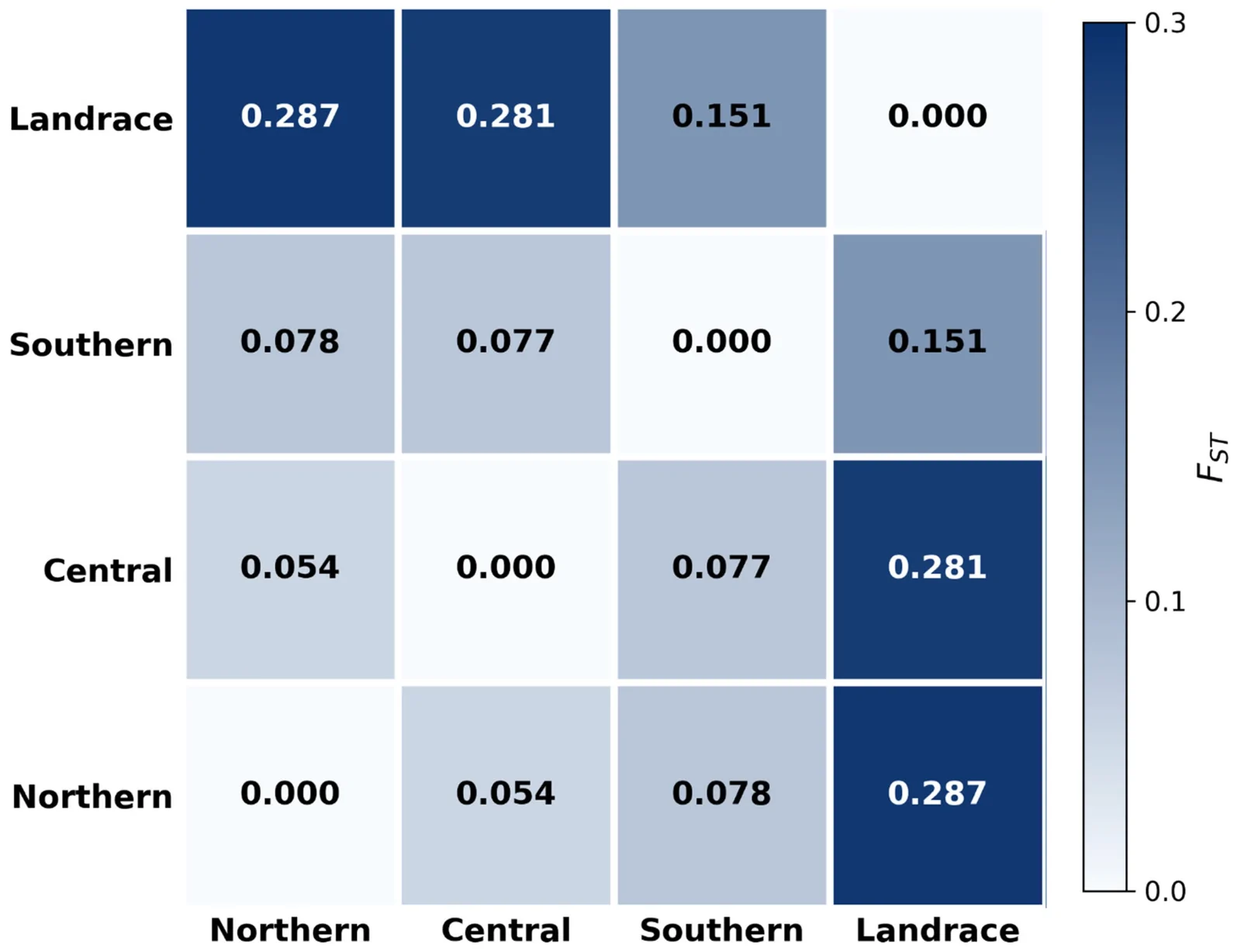
Mean pairwise FST between regional pig groups. Vietnamese vs. Landrace (FST = 0.151–0.287) are substantially higher than within-Vietnamese comparisons (FST = 0.054–0.078). Diagonal values are zero.

**Figure 3:**
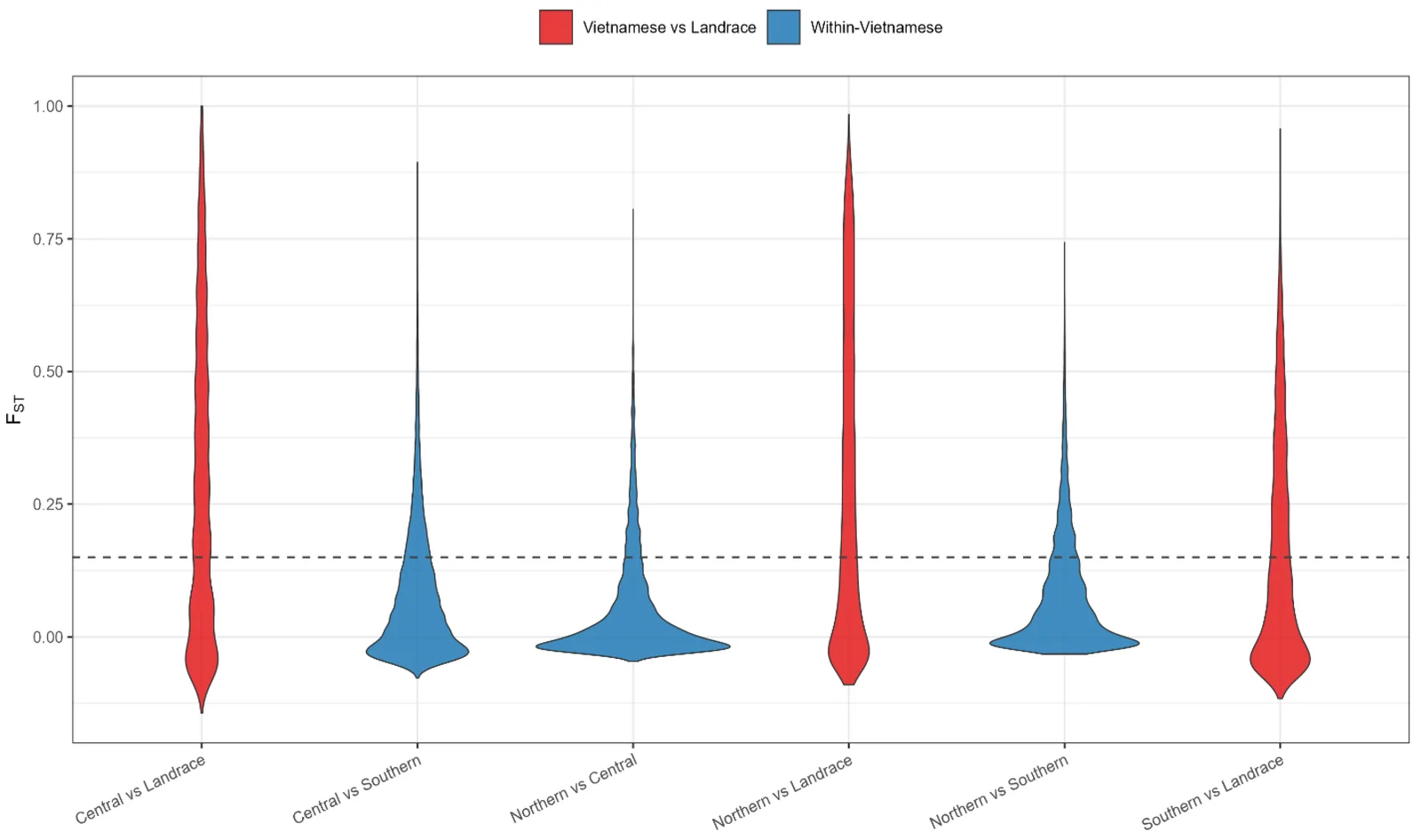
Distribution of per-SNP FST values across all pairwise comparisons. Vietnamese vs. Landrace comparisons (red) are right-shifted relative to within-Vietnamese comparisons (blue). Dashed vertical line at FST = 0.15 for reference.

**Figure 4:**
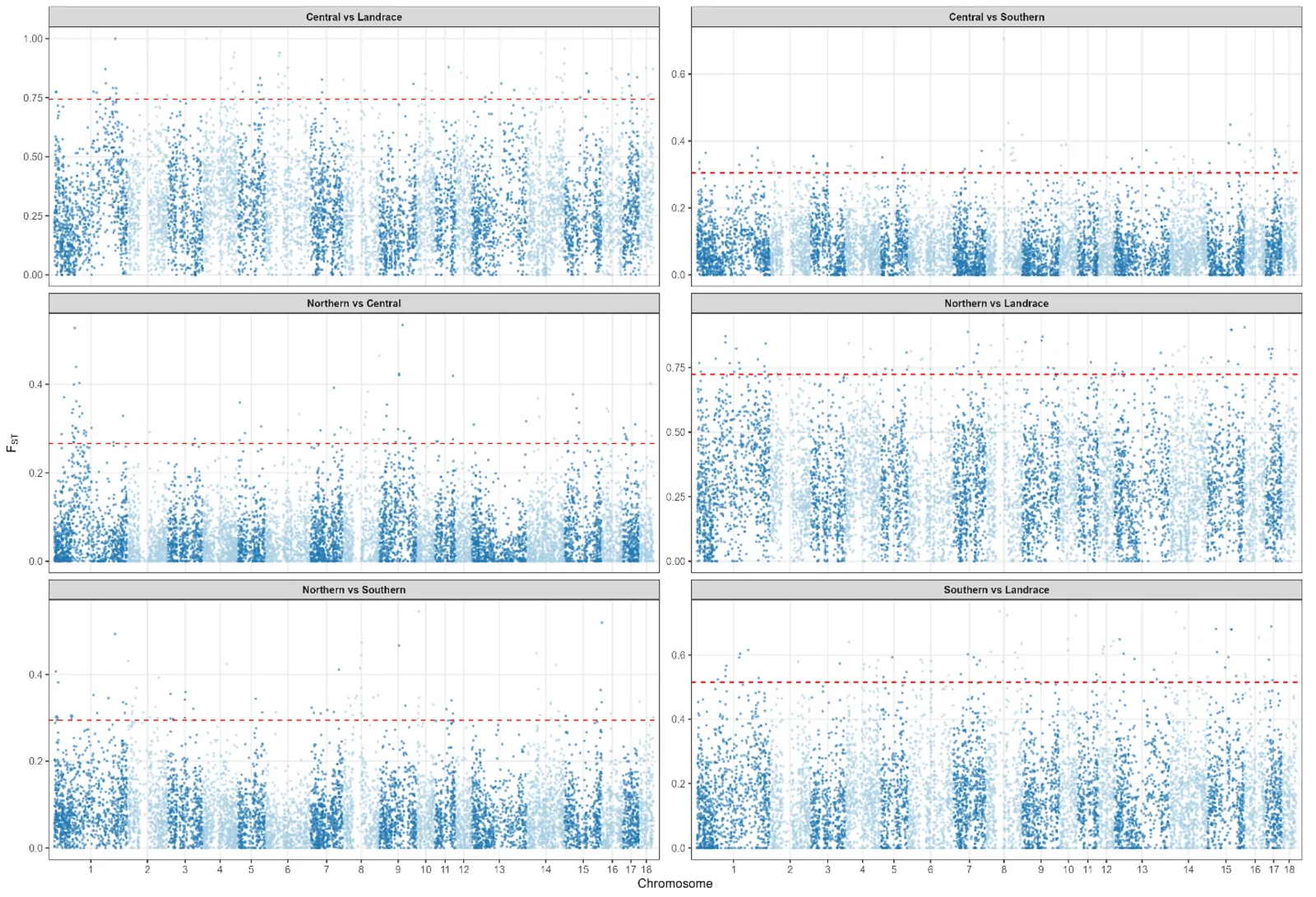
Genome-wide windowed FST (100 kb) for all regional pairwise comparisons. Each point = one window. Red dashed = 99th percentile. Alternating blue shades distinguish chromosomes.

Vietnamese regional groups showed strong differentiation from Landrace (mean FST = 0.151–0.287) and moderate within-Vietnamese differentiation (mean FST = 0.054–0.078; Table 2, Figure 2). Northern and Central breeds showed the highest differentiation from Landrace (FST = 0.286 and 0.280, respectively), while Southern breeds showed markedly lower FST vs. Landrace (FST = 0.151), providing suggestive evidence of a European genetic contribution to this group. It is important to note that FST quantifies genetic differentiation rather than admixture proportion, so this contrast is not in itself evidence of introgression. Within-Vietnamese comparisons ranged from FST = 0.054 (Northern vs. Central) to FST = 0.078 (Northern vs. Southern). The distribution of per-SNP FST values across all six pairwise comparisons is shown in Figure 3. Per-comparison SNP counts in Table 2 range from 47,085 to 48,746, slightly below the genome-wide total of 48,896, because PLINK excludes markers with insufficient genotype data in either group for a given pairwise comparison. Because the Central group comprises only two breeds (*n* = 12), per-SNP FST at markers fixed differently between groups can approach the theoretical maximum of 1.0; this reflects the small sample size rather than necessarily indicating exceptionally strong selection. Windowed FST (Figure 4), a weighted mean of per-SNP FST within each 100 kb window, is consequently smaller than these per-SNP maxima.

**Table 2:** Summary of pairwise FST estimates between regional groups.

Comparison	N SNPs	Mean FST	Median FST	Max FST	99th pct
Northern vs. Landrace	48,689	0.287	0.217	0.984	0.884
Central vs. Landrace	47,085	0.281	0.227	1	0.94
Southern vs. Landrace	48,319	0.151	0.089	0.957	0.687
Northern vs. Southern	48,746	0.078	0.04	0.743	0.459
Central vs. Southern	48,030	0.077	0.039	0.894	0.516
Northern vs. Central	48,113	0.054	0.013	0.805	0.438

### 2.4. Windowed FST and XP-EHH Selection Signals

**Figure 5:**
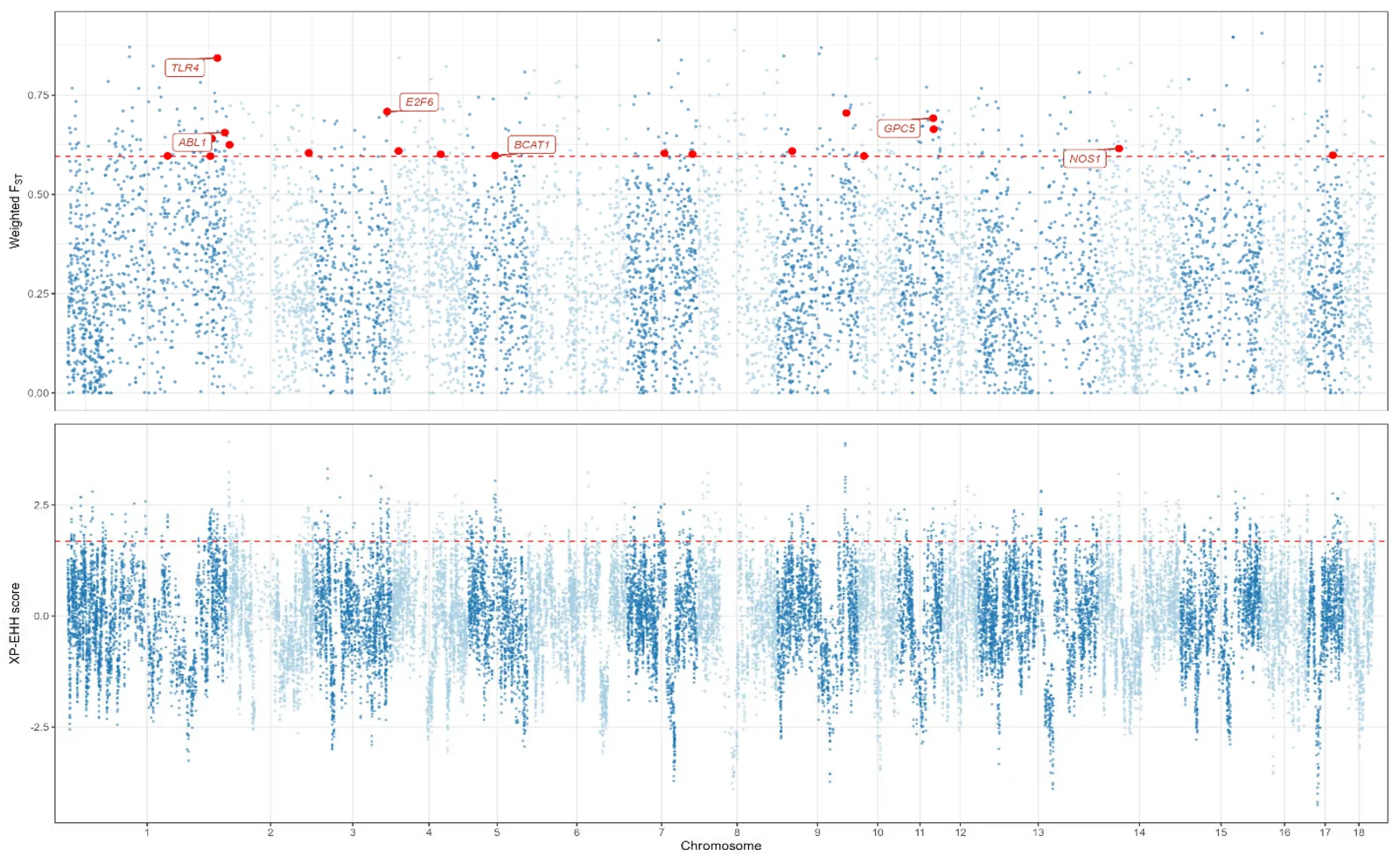
Integrated selection signals for a pair comparison between Northern Vietnamese breeds and Landrace. Upper panel: genome-wide windowed FST (100 kb windows); red filled points = high-confidence windows in top 5% of both FST and XP-EHH simultaneously (*n* = 20 windows). Lower panel: XP-EHH scores. Red dashed lines = 95th percentile thresholds. Key candidate genes are annotated at peak windows: *TLR4* (Chr1:258.0 Mb; FST = 0.843), *E2F6* (Chr3:125.2 Mb; FST = 0.709), *GPC5* (Chr11:61.5 Mb; FST = 0.692), *ABL1* (Chr1:270.8 Mb; FST = 0.656), *NOS1* (Chr14:35.2 Mb; FST = 0.615; XP-EHH = 3.20), *BCAT1* (Chr5:48.8 Mb; FST = 0.598). The combined two-panel visualization shows that high-confidence windows exceed the 95th percentile of both elevated FST and extended haplotype homozygosity; because both statistics were computed from the same genotypes, this represents analytical concordance rather than independent support. Red dots without labels indicate high-confidence windows containing no annotated protein-coding gene or only uncharacterized loci.

Windowed FST (Figure 4) identified numerous differentiation hotspots between Vietnamese breeds and Landrace, with a mean windowed FST of 0.280–0.286 for Vietnamese vs. Landrace and 0.054–0.078 for within-Vietnamese comparisons. Integration of FST and XP-EHH (top 5% in both methods simultaneously) identified 20 high-confidence windows in Northern vs. Landrace and 3 in Central vs. Landrace, with zero shared windows (Table 3, Figure 5). Threshold sensitivity analysis identified 102 windows at the 90th percentile and zero at the 99th percentile; *TLR4*, *GPC5*, *E2F6*, *NOS1*, *ABL1*, *BCAT1*, and *ABCA1* were consistently identified at both the 90th and 95th percentile thresholds (Supplementary Table S2). Because all thresholds were applied to the same individuals and the same SNP dataset, this consistency indicates analytical robustness to threshold choice rather than independent biological validation. The 99th percentile returned zero windows, consistent with expected behavior at *n* = 6 per breed and medium-density SNP data. Top Northern candidate loci: *TLR4* (Chr1:258.0 Mb; FST = 0.843), *E2F6* (Chr3:125.2 Mb; FST = 0.709), *GPC5* (Chr11:61.5 Mb; FST = 0.692). Top Central candidate loci: *ZP2*/*LDAF1*/*CRYM*/*ANKS4B* (Chr3:24.8 Mb; FST = 0.614), *BRIX1*/*RAD1*/*AGXT2* (Chr16:20.5 Mb; FST = 0.704).

**Table 3:** High-confidence genomic windows in top 5% of both windowed FST and XP-EHH in Northern and Central Vietnamese breeds vs. Landrace *.

Comp.	Chr	Position (Mb)	Win FST	Max XP-EHH	Key Candidate Gene(s)	Function
N vs. L	1	258.0	0.843	1.86	*TLR4*	Toll-like receptor 4; innate immune signaling
N vs. L	3	125.2	0.709	1.99	*E2F6*	Cell cycle TF; antiviral immune
N vs. L	9	119.1	0.705	2.00	—	No annotated gene
N vs. L	11	61.5	0.692	2.10	*GPC5*	Glypican-5; IGF/FGF/Wnt signaling
N vs. L	11	62.3	0.664	1.94	—	No annotated gene
N vs. L	1	270.8	0.656	2.03	*ABL1*	Tyrosine kinase; immune signaling
N vs. L	1	248.4	0.641	1.84	*RAD23B*	DNA damage recognition; nucleotide excision repair
N vs. L	2	5.0	0.625	2.03	*CABP2*, *CDK2AP2*, *PITPNM1*	Calcium binding; cell signaling
N vs. L	14	35.2	0.615	3.20	*NOS1*	Nitric oxide synthase; immune/stress
N vs. L	4	12.9	0.609	2.06	—	No annotated gene
N vs. L	9	25.4	0.609	1.87	—	No annotated gene
N vs. L	2	141.9	0.605	2.21	*CYSTM1*	Cysteine-rich transmembrane
N vs. L	7	65.6	0.604	2.52	—	No annotated gene
N vs. L	4	85.7	0.601	1.76	—	No annotated gene
N vs. L	7	114.0	0.601	1.81	*SLC24A4*, *RIN3*	Solute carrier; mineral transport
N vs. L	17	43.4	0.599	2.76	*MAFB*	TF; myeloid differentiation
N vs. L	5	48.8	0.598	3.05	*BCAT1*	Branched-chain AA catabolism
N vs. L	1	172.1	0.597	2.13	*LRFN5*	Neuronal cell adhesion
N vs. L	10	10.2	0.597	1.68	*MTARC1*, *HLX*	Homeobox TF; developmental
N vs. L	1	246.1	0.596	2.07	*NIPSNAP3B*, *ABCA1*	Cholesterol/lipid transport
C vs. L	16	20.5	0.704	1.98	*BRIX1*, *RAD1*, *AGXT2*	Ribosome biogenesis; DNA repair; amino acid metabolism
C vs. L	3	24.8	0.614	2.53	*ZP2*, *LDAF1*, *CRYM*, *ANKS4B*	Thyroid hormone binding; zona pellucida; lipid droplet
C vs. L	15	58.5	0.612	1.98	—	No annotated gene

*: Win FST = windowed FST for the relevant comparison; Max XP-EHH = maximum windowed XP-EHH in the high-confidence window. N vs. L = Northern vs. Landrace; C vs. L = Central vs. Landrace. High-confidence = top 5% in both FST and XP-EHH simultaneously. Zero windows were shared between Northern and Central sets, indicating independent selection histories. — = no annotated protein-coding genes.

### 2.5. Inbreeding Analysis (ROHs)

**Figure 6:**
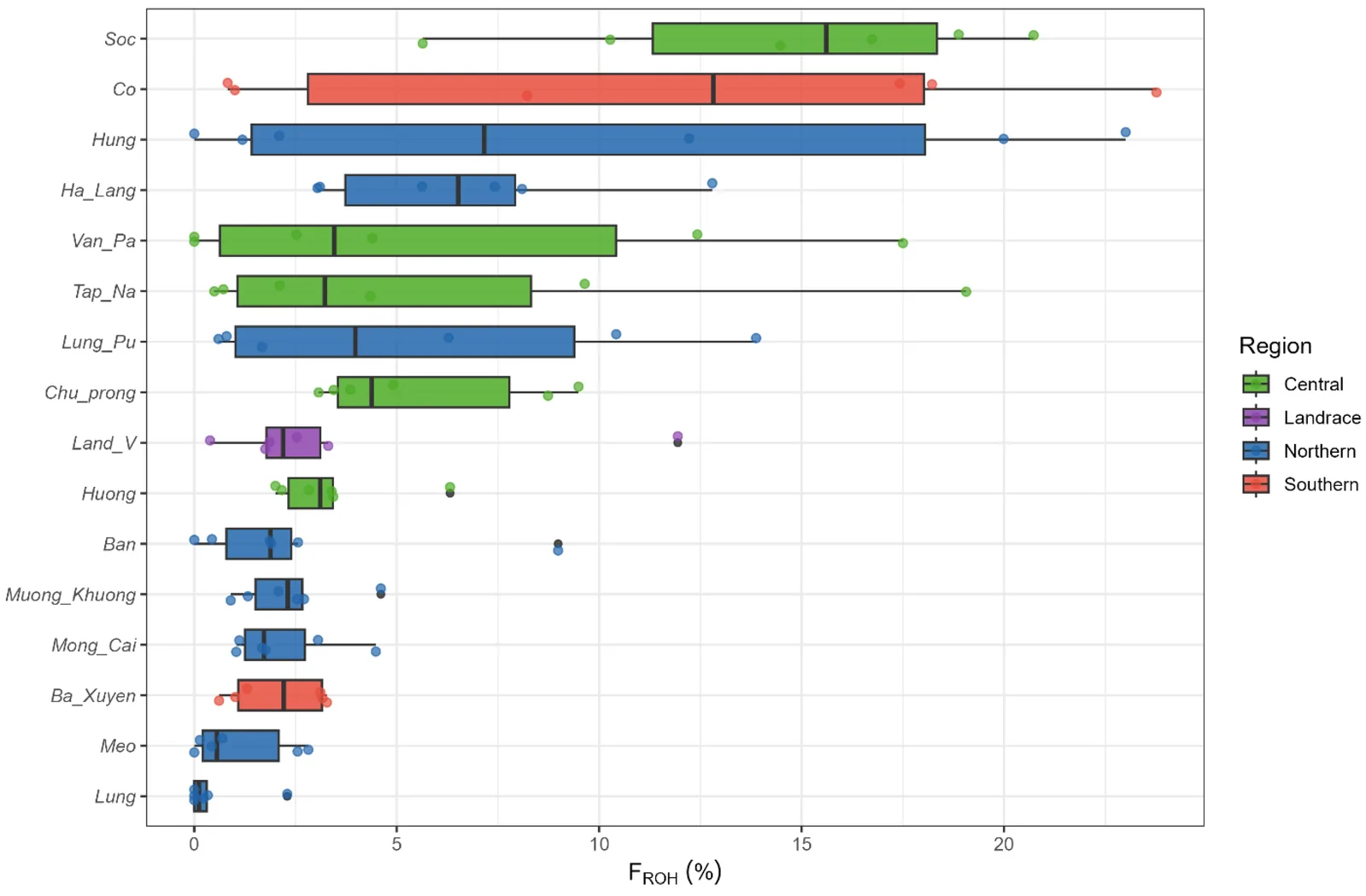
Inbreeding coefficient (FROH) by breed, computed as total ROH length (minimum 1 Mb) divided by autosomal genome length (2472 Mb; Sscrofa11.1). Breeds ordered by mean FROH within each geographic region (Northern = Ban, Meo, Hung, Muong_Khuong, Ha_Lang, Lung_Pu, Lung, Mong_Cai, Tap_Na, Huong; Central = Co, Van_Pa; Southern = Ba_Xuyen, Soc, Chu_prong). Soc (Southern; FROH = 14.46%), Co (Central; FROH = 11.58%), and Hung (Northern; FROH = 9.75%) show the highest autozygosity and are therefore identified as breeds warranting particular attention in conservation planning; autozygosity alone does not establish conservation priority. Ba_Xuyen shows low FROH consistent with a recent European genetic contribution. Individual data points shown as jittered dots; box = interquartile range; line = median.

FROH ranged from 0.48% (Lung) to 14.46% (Soc), a >30-fold range (Table 4, Figure 6). Soc, Co, and Hung showed the highest inbreeding (FROH = 14.46%, 11.58%, and 9.75%, respectively), while Lung and Meo showed the lowest (FROH = 0.48% and 1.10%). Ba Xuyen showed low FROH (2.08%) despite geographic proximity to Co (11.58%), consistent with heterozygosity introduced through a European genetic contribution. Landrace showed intermediate FROH (3.63%).

**Table 4:** ROHs and FROH summary by breed following geographic region assignments.

Region	Breed	N	Mean FROH (%)	SD FROH	Mean ROHs (Mb)	Mean N ROHs
Southern	Soc	6	14.46	5.65	357.4	21.0
	Chu_prong	6	5.58	2.81	138.1	15.5
	Ba_Xuyen	6	2.08	1.23	51.4	6.5
Central	Co	6	11.58	9.65	286.2	14.2
	Van_Pa	6	6.14	7.21	151.8	7.8
Northern	Hung	6	9.75	10.13	241.0	12.7
	Ha_Lang	6	6.68	3.66	165.1	11.8
	Tap_Na	6	6.06	7.21	149.9	9.2
	Lung_Pu	6	5.61	5.58	138.6	9.3
	Huong	6	3.36	1.57	83.0	7.2
	Ban	6	2.62	3.26	64.9	5.3
	Muong_Khuong	6	2.36	1.30	58.3	7.2
	Mong_Cai	6	2.19	1.34	54.1	6.3
	Meo	6	1.10	1.25	27.3	3.3
	Lung	6	0.48	0.90	11.8	1.3
Landrace	Landrace	6	3.63	4.18	97.5	10.8

A notable result in this study was the heterogeneity in ROH length class composition across Vietnamese breeds. Long ROHs (>8 Mb), indicative of recent inbreeding within the past approximately 6–12 generations, dominated the total autozygosity in all breeds, accounting for the majority of FROH in Soc (~330 Mb long ROHs per individual), Co (~245 Mb), Hung (~230 Mb), Ha_Lang (~165 Mb), Van_Pa (~155 Mb), and Tap_Na (~145 Mb). In contrast, Meo and Lung showed the lowest total ROHs of any Vietnamese breed across all length classes, with long ROHs contributing fewer than 15 Mb per individual on average, consistent with their status as relatively outbred highland populations. Ba_Xuyen showed disproportionately low long ROHs relative to total autozygosity compared to other Southern breeds, consistent with the suggestive evidence of a European genetic contribution to this breed obtained from PCA and heterozygosity, which the f3 test did not formally support. Short ROHs (1–4 Mb) were negligible across all breeds, suggesting the absence of detectable ancient bottleneck signatures within the resolution of the 60K SNP array.

### 2.6. Within-Vietnamese Comparisons Between Breed-Level Phenotypic Groups

Within-Vietnamese phenotypic XP-EHH analysis comparing lean small-bodied breeds (*n* = 42; Ban, Hung, Lung, Van_Pa, Co, Soc, Chu_prong; predominantly black-coated) against three contrasting breed groups identified genomic windows differentiating these groups. Breeds were classified into three phenotypic groups based on Nguyen et al. (2026) [2]: lean/small-bodied (*n* = 42), extreme fat/prolific (*n* = 18: Mong_Cai, Huong, Ha_Lang; white/light-coated, large-bodied), and medium-bodied (*n* = 24: Muong_Khuong, Lung_Pu, Tap_Na, Meo). Individual-level measurements of body weight, backfat thickness, fat percentage, carcass traits, and litter size were not available for these animals. The groupings are therefore breed-level categories derived from published breed descriptions and coat color, and this analysis quantifies genomic differentiation among breeds classified into phenotype-related groups rather than genetic association with individual body-composition phenotypes. All results in this section should accordingly be interpreted as descriptive and exploratory breed-level comparisons. A candidate genomic region was identified at Chr14:59.5–62.5 Mb, containing genes with convergent roles in fat deposition and skeletal muscle structure and distinguishing breeds with contrasting body composition profiles. *GALNT2* (Chr14:60.05 Mb) showed consistent XP-EHH signals across all three comparisons (lean vs. extreme XP-EHH = 2.50; lean vs. medium XP-EHH = 4.93; lean vs. prolific XP-EHH = 4.52), encoding a major QTL for lipid metabolism and HDL cholesterol. *BICC1* (Chr14:61.96 Mb) showed the same profile with a stronger lean vs. extreme signal (lean vs. extreme XP-EHH = 4.21; lean vs. medium 3.34; lean vs. prolific 4.03), encoding an RNA-binding protein involved in post-transcriptional regulation. *CAPN9*, *ACTA1* and *RHOU*, previously reported as co-located candidates in this region, did not clear the 95th percentile threshold in any phenotypic comparison in the corrected analysis. Notably, GALNT2 did not reach the top 5% FST threshold in any pairwise regional comparison (max FST = 0.259), consistent with differentiation at this locus arising among Vietnamese breed groups rather than between Vietnamese breeds and the sampled Landrace population.

The phenotypic XP-EHH analysis identified three classes of candidates based on their comparison-specific profiles. First, candidates shared across breed groups showed signals in all comparisons involving the lean group: *GALNT2* (Chr14:60.05 Mb) was present in lean vs. extreme (XP-EHH = 2.50), lean vs. medium (XP-EHH = 4.93), and lean vs. prolific (XP-EHH = 4.52) but did not exceed the 95th percentile threshold in the medium vs. extreme comparison (Max XP-EHH = 0.48), indicating that it differentiates the larger-bodied breed groups from the lean breed group irrespective of fat category; *BICC1* (Chr14:61.96 Mb) shows a comparable profile (4.21/3.34/4.03), although its medium vs. extreme value (1.72) coincides with the 95th percentile threshold for that comparison (1.717), so its assignment between the shared and the extreme-fat/prolific classes is not resolved by these data. Second, candidates specific to the extreme-fat/prolific breed group were identified by above-threshold signals in both lean vs. extreme and medium vs. extreme: *HCAR1* (Chr14:30.1 Mb; lean vs. extreme XP-EHH = 3.79; medium vs. extreme XP-EHH = 2.60) and *ATG10* (Chr2:90.55 Mb; lean vs. extreme XP-EHH = 4.42; medium vs. extreme XP-EHH = 1.94), indicating that lean and medium breeds share similar haplotypes at these loci while extreme fat/prolific breeds carry distinct haplotypes. Third, candidates specific to the medium-bodied breed group showed signals only in the lean vs. medium comparison: *EFNA5* (Chr2:112.68 Mb; XP-EHH = 2.00) and *HIPK2* (Chr18:10.00 Mb; XP-EHH = 3.25). The *WNT16*/*FAM3C* region (Chr18:25.4–25.6 Mb) cleared the threshold in lean vs. extreme (1.84) and lean vs. prolific (2.21) but not lean vs. medium (1.33). The genome-wide XP-EHH signal for the lean vs. extreme comparison is shown in Figure S3, panel A, and summary statistics for the four comparisons in Supplementary Table S9.

The extreme-fat/prolific-group candidates HCAR1 and ATG10 both showed above-threshold signals in the medium vs. extreme comparison (Figure S3). *HCAR1* (Chr14:30.1 Mb; lean vs. extreme XP-EHH = 3.79; medium vs. extreme XP-EHH = 2.60; Central vs. Landrace FST = 0.647) encodes hydroxycarboxylic acid receptor 1, a lactate receptor in adipose tissue that directly suppresses lipolysis. Its signal above the 95th percentile in both lean vs. extreme and medium vs. extreme indicates that the lean and medium-bodied breed groups share similar haplotypes while the extreme-fat/prolific breeds (Mong_Cai, Huong, Ha_Lang) carry a distinct haplotype. Selection for reduced lipolysis is therefore a hypothesis generated by this observation rather than a demonstrated mechanism, and *HCAR1* remains a genomic candidate that has not been functionally validated. *ATG10* (Chr2:90.55 Mb; lean vs. extreme XP-EHH = 4.42; medium vs. extreme XP-EHH = 1.94; Central vs. Southern FST = 0.323) encodes an E2-like conjugating enzyme essential for autophagosome formation; autophagy directly regulates lipid droplet degradation in adipose tissue, and the convergent XP-EHH and FST signals at this locus are consistent with, but do not demonstrate, selection on autophagy-mediated fat mobilization as a second axis of differentiation between these breed groups. *HIPK2* (Chr18:10.00 Mb; lean vs. medium XP-EHH = 3.25; Central vs. Landrace FST = 0.595) was identified as a candidate specific to the medium-bodied breed group by both XP-EHH and FST analyses, encoding a kinase regulating adipogenesis, and did not reach threshold in the medium vs. extreme comparison (XP-EHH = −0.20). *EFNA5* (Chr2:112.68 Mb; lean vs. medium XP-EHH = 2.00; stable across Ha_Lang reclassification) encodes ephrin-A5, a cell adhesion molecule expressed in adipose tissue that regulates adipocyte differentiation. The *WNT16*/*FAM3C*/*CPED1* region (Chr18:25.4–25.6 Mb; lean vs. extreme XP-EHH = 1.84; lean vs. prolific XP-EHH = 2.21; lean vs. medium XP-EHH = 1.33; medium vs. extreme XP-EHH = 1.20) showed a body frame size signal between lean and extreme/prolific breeds but not between lean and medium-bodied breeds (Table 5, Figure S3).

### 2.7. Haplotype Bifurcation at High-Confidence Candidate Loci

At the GPC5 locus (Chr11:61,553,605), Northern Vietnamese breeds (*n* = 60 individuals, 120 haplotypes) carried the ancestral allele (Figure S2) exclusively. This allele formed a single dense haplotype trunk extending across approximately 50 Mb in both directions from the focal SNP with minimal branching, indicating near-complete fixation of a single ancestral haplotype, a haplotype pattern consistent with recent positive selection; the present data cannot distinguish this pattern from fixation by drift in a small sample. In contrast, Landrace haplotypes at the same locus showed both ancestral and derived alleles with immediate branching, reflecting normal background recombination; given the small Landrace sample size (*n* = 6), this pattern is consistent with, but does not conclusively establish, the absence of recent selection at this locus in European commercial pigs.

### 2.8. GO and Pathway Enrichment of Selection Candidate Genes

**Figure 7:**
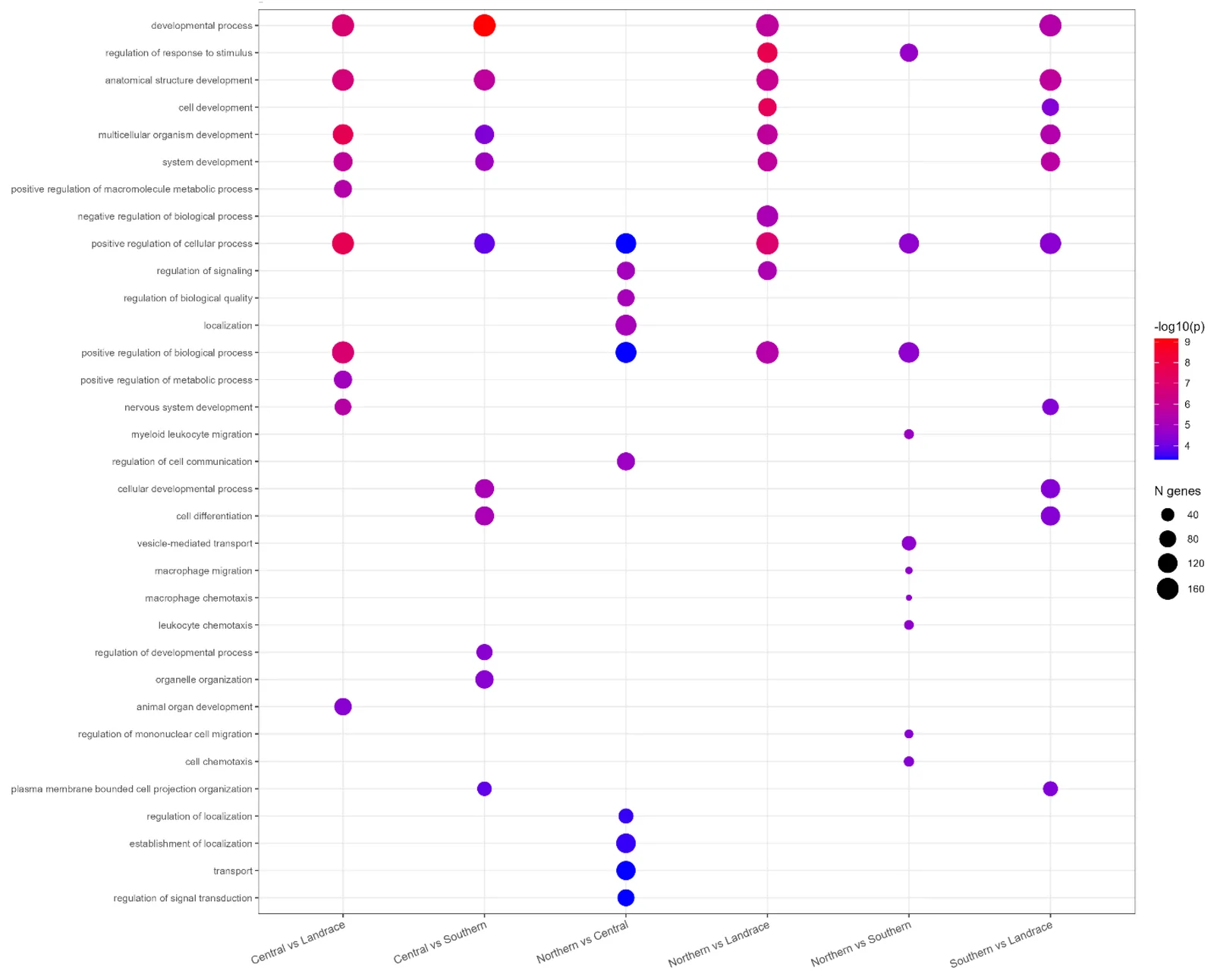
Combined GO Biological Process (GO:BP) enrichment dot plot across all six pairwise regional comparisons (FDR < 0.05, g:Profiler sscrofa annotation). Dot size = gene ratio (overlapping candidate genes/total genes in GO term); color = −log10 *p*-value (red = most significant, blue = near FDR threshold). Northern vs. Landrace shows the strongest and broadest enrichment (349 GO:BP terms). Northern vs. Southern shows specific enrichment for myeloid leukocyte migration and macrophage chemotaxis, consistent with differential immune selection pressure between highland Northern and lowland Southern breeds. Full GO:MF, GO:CC, KEGG, and Reactome results are provided in the Supplementary Tables.

GO enrichment analysis identified significant terms (FDR < 0.05) across all six pairwise comparisons, with GO:BP terms dominating the results (Figure 7; Supplementary Tables S5–S8). The strongest enrichment was observed in the Northern vs. Landrace comparison (349 GO:BP terms), followed by Northern vs. Central (118 terms), Central vs. Landrace (111 terms), and Southern vs. Landrace (106 terms). The two within-Vietnamese comparisons involving Southern breeds yielded the fewest terms—Central vs. Southern (90 terms) and Northern vs. Southern (89 terms). The Northern vs. Southern comparison showed specific enrichment for myeloid leukocyte migration (17 genes; *p* = 1.46 × 10^−5^), leukocyte chemotaxis (16 genes; *p* = 2.94 × 10^−5^) and macrophage chemotaxis (8 genes; *p* = 2.94 × 10^−5^).

The three Vietnamese vs. Landrace comparisons shared a common core of enriched biological processes, including anatomical structure development, cell development, multicellular organism development, and developmental process (FDR < 0.05), reflecting the deep evolutionary divergence between Asian and European pig lineages and consistent with selection on growth trajectories and body composition during domestication. The Northern vs. Landrace comparison additionally showed strong enrichment for regulation of response to stimulus (*p* = 1.95 × 10^−8^) and cell surface receptor signaling pathway, consistent with selection on environmental sensing and stress response in Northern Vietnamese highland breeds.

A notable result in this study was the identification of biologically coherent KEGG and Reactome pathways enriched among candidate genes in top 5% windowed FST windows following breed assignment correction. In the Northern vs. Central comparison, g:Profiler identified the Type II diabetes mellitus pathway (KEGG; 8 genes; adjusted *p* = 3.45 × 10^−3^), with co-located *HKDC1*/*HK1* (Chr14:72.3 Mb; FST = 0.266), *ABCC8*/*KCNJ11* (Chr2:41.7 Mb; FST = 0.219), *IRS1* (Chr15; FST = 0.249), and *MTOR* (Chr6; FST = 0.235), implicating glucose metabolism and insulin secretion in the divergence between Northern highland and Central coastal breeds (Table 6). In the Northern vs. Southern comparison, g:Profiler identified two Reactome pathways: CDC42 GTPase cycle (adjusted *p* = 0.030) and HS-GAG degradation (adjusted *p* = 0.033). Supplementary clusterProfiler analysis additionally identified four KEGG pathways in the Northern vs. Southern comparison (all BH-adjusted *p* < 0.05): viral protein interaction with cytokine and cytokine receptor (8 genes; BH-adjusted *p* = 0.036), Pertussis (7 genes; BH-adjusted *p* = 0.038), PI3K-Akt signaling pathway (16 genes; BH-adjusted *p* = 0.038), and Cadherin signaling (17 genes; BH-adjusted *p* = 0.038). A common core of innate immune genes drove enrichment across multiple Northern vs. Southern pathways: *CXCL8* (Chr8; FST = 0.445), *TLR4* (Chr1; FST = 0.336), *RELA* (Chr2; FST = 0.282), and *CCR1*/*CCR2* (Chr13; FST = 0.219). With the corrected panel, Central vs. Southern showed enrichment for viral protein interaction with cytokine and cytokine receptor (9 genes; BH-adjusted *p* = 0.036), Southern vs. Landrace for African trypanosomiasis (6 genes; BH-adjusted *p* = 0.043), plus two Reactome synaptic transmission pathways (adjusted *p* = 0.037) and no KEGG or Reactome pathway reached FDR < 0.05 for Central vs. Landrace (Table 6).

### 2.9. Within-Population iHS Analysis

iHS analysis within each regional group was used to assess whether the XP-EHH signals were also supported by within-population haplotype structure. It is important to note that iHS and XP-EHH were computed from the same individuals and the same SNP dataset; agreement between them therefore indicates internal analytical consistency and cannot be interpreted as independent biological replication. *E2F6* (Chr3:125.1 Mb) showed strong Northern-specific iHS (|iHS| = 3.04) with weaker signals in Central (|iHS| = 1.22) and Southern (|iHS| = 1.89) breeds, consistent with a candidate Northern-specific sweep signal. *NOS1* (Chr14:35.3 Mb) showed strong iHS across all three groups (Northern 3.18, Central 2.66, Southern 3.36), consistent with a candidate pan-Vietnamese selection signal at this locus. *GALNT2* (Chr14:60.0 Mb) showed strong iHS in Northern (|iHS| = 3.01) and Southern (|iHS| = 2.48) breeds but not Central (|iHS| = 1.64), consistent with its classification as a candidate shared across breed groups. *HCAR1* (Chr14:30.1 Mb) showed the strongest iHS in Northern breeds (|iHS| = 3.70), with a weaker signal in Central (|iHS| = 2.56) and none above threshold in Southern breeds (|iHS| = 1.58); its assignment to the extreme-fat/prolific breed group therefore rests on the XP-EHH evidence rather than on regional iHS specificity. *GPC5* (Chr11:61.5 Mb) showed moderate iHS in Southern breeds (|iHS| = 2.39) but not Northern (|iHS| = 1.28), suggesting a candidate sweep signal at this locus in Southern breeds. iHS Manhattan plots for all three regional groups are shown in Figure S4. The f3 results for all tested breeds are provided in Supplementary Table S3; iHS values at key loci in Supplementary Table S4; and threshold sensitivity results in Supplementary Table S2.

## 3. Discussion

### 3.1. Population Structure and Evidence for a European Genetic Contribution to Ba Xuyen

PCA and FST analyses are consistent with deep divergence between Vietnamese and European pig lineages and identify Ba Xuyen as a breed showing suggestive evidence of a European genetic contribution. Ba Xuyen showed markedly elevated observed heterozygosity (0.388 vs. 0.164–0.366 for local Vietnamese breeds) and clustered intermediately between Landrace and Southern breeds on PC1. To formally test admixture, we computed f3(Ba_Xuyen; Landrace, Vietnamese_reference) = +0.015 (Z = +39.9). The positive f3 did not meet the formal criterion for admixture, which requires a significantly negative f3 (f3 < 0 with Z < −3), and the present analysis therefore provides no formal statistical support for admixture in Ba Xuyen. However, it is not clear how strongly the known sensitivity of f3 to small sample sizes (*n* = 6) affects this estimate, but we speculate that it might have influenced the outcome of our results. Therefore, the convergent evidence from PCA, FST, and heterozygosity is suggestive evidence of a European genetic contribution to Ba Xuyen rather than as confirmed European admixture, and formal admixture analysis in larger cohorts is required before this interpretation can be regarded as confirmatory.

### 3.2. Regional Candidate Genes: Asian–European Divergence

Among the high-confidence selection windows (20 Northern, 3 Central vs. Landrace; zero shared), TLR4 (Chr1:258.0 Mb; FST = 0.843) shows the highest windowed FST in the dataset, encoding toll-like receptor 4, a pattern-recognition receptor central to innate immune detection of bacterial pathogens; this converges with the TLR4 signal in the Northern vs. Southern immune enrichment (Section 3.6); because both analyses were derived from the same individuals and the same genotype dataset, this convergence shows cross-method consistency rather than independent support. The sole surviving Central vs. Landrace candidate, *CRYM*/*ZP2* (Chr3:24.8 Mb; FST = 0.614), lies in a region also containing *LDAF1* and *ANKS4B*; given the small Central group (*n* = 12), this signal should be treated as exploratory. *GPC5* (glypican−5, Chr11) remains biologically meaningful, modulating IGF, FGF, and Wnt signaling [15,16,17]. *E2F6* (Chr3:125.2 Mb; FST = 0.709) was also supported by iHS (Northern |iHS| = 3.04 vs. Central 1.22 and Southern 1.89). Because iHS and XP-EHH were derived from the same individuals and the same SNP dataset, this concordance provides cross-method rather than independent support, and *E2F6* is best described as a candidate locus for a Northern-specific sweep. E2F6 has documented roles in antiviral transcriptional repression in other species, suggesting this signal might reflect selection on immune-related, rather than purely morphological, traits; however, this interpretation remains speculative without functional validation. *NOS1* (Chr14:35.2 Mb; XP-EHH = 3.69) showed strong iHS across all three regional groups (|iHS| = 2.66–3.36), consistent with pan-Vietnamese selection at this locus, although neither the timing of any such selection nor its relationship to immune function can be established from the present data. *ABL1* (Chr1:270.8 Mb; FST = 0.656) encodes a tyrosine kinase involved in immune and stress signaling. These results are in agreement with published selection signature studies reporting immune gene enrichment in highland-adapted Asian pig populations [8]. It is important to note that all genes discussed in this section are genomic candidates identified from public annotation of the regions concerned; none were functionally validated in this study, and the functional interpretations offered here are therefore hypotheses requiring experimental confirmation.

### 3.3. Within-Vietnamese Diversification: Pigmentation, Body Size, and Metabolism

The within-Vietnamese analysis describes genomic differentiation between lean black-coated breeds and larger white or light-coated breeds across both the extreme-fat/prolific and the medium-bodied breed groups. It is important to note that these groups were assigned from published breed descriptions and coat color rather than from individual-level body-composition measurements, so the following interpretations concern differentiation among phenotype-related breed groups rather than genotype–phenotype associations at the individual level. The Chr14:59.5–62.5 Mb region emerges as a candidate genomic region differentiating breeds classified into contrasting phenotype-related groups, with *GALNT2* showing the most consistent signal across all phenotypic comparisons. *GALNT2* is a well-established lipid metabolism QTL that regulates HDL cholesterol and triglyceride levels [18,19], and its colocalization with *CAPN9* (muscle-specific calpain), *ACTA1* (skeletal muscle alpha-actin), and *RHOU* (adipocyte Rho GTPase) may be consistent with selection acting on muscle composition and fat deposition, although neither trait was measured in these animals. These results are in agreement with QTL studies in commercial pig breeds identifying Chr14 as a major body composition region [20].

*HCAR1* and *ATG10* were further supported as candidates specific to the extreme-fat/prolific breed group by their signal above the 95th percentile threshold in both the lean vs. extreme and medium vs. extreme comparisons. This represents the most consistent within-study evidence for differentiation associated with the extreme-fat/prolific breed group of Mong_Cai, Huong, and Ha_Lang; because both comparisons draw on the same genotypes and because the phenotype is assigned at breed level, this consistency is analytical rather than independent biological evidence. *HCAR1* (Chr14:30.1 Mb) showed the strongest iHS in Northern breeds (|iHS| = 3.70), a weaker Central signal (|iHS| = 2.56) and no above-threshold Southern signal (|iHS| = 1.58); the assignment of *HCAR1* to the extreme-fat/prolific breed group therefore rests on the XP-EHH evidence rather than on iHS being confined to one region. *HCAR1* also appeared in the Central vs. Landrace FST comparison (FST = 0.647), providing cross-method convergence within the same dataset. *ATG10* (Chr2:90.55 Mb) appeared in both Central vs. Southern (FST = 0.323) and Northern vs. Southern (FST = 0.216) regional comparisons, suggesting that selection on autophagy-mediated fat mobilization also contributed to regional genomic divergence across Vietnam’s geographic zones. The biological convergence of *HCAR1* (lactate-mediated lipolysis suppression) and *ATG10* (autophagy-mediated lipid droplet degradation) as candidates specific to the extreme-fat/prolific breed group suggests these genes may represent candidate loci for two complementary fat-retention mechanisms [21,22], though this interpretation requires functional validation before these can be considered defining features of the extreme fat/prolific phenotype. In contrast, *GALNT2* showed strong iHS in Northern (|iHS| = 3.01) and Southern (|iHS| = 2.48) but not Central (|iHS| = 1.64) breeds, corroborating its classification as a candidate shared across breed groups. *GALNT2* did not exceed the threshold in the medium vs. extreme comparison (XP-EHH = 0.48), consistent with its classification as a candidate shared across all larger-bodied Vietnamese breed groups. The signals specific to the medium-bodied breed group at *EFNA5* (Chr2:112.68 Mb) and *HIPK2* (Chr18:10.00 Mb) were absent from the medium vs. extreme comparison, consistent with their potential role in moderate body size [23,24] divergence rather than extreme fat deposition. Further studies on *HCAR1* and *ATG10* expression in Vietnamese pig adipose tissue, together with individual-level body-composition phenotyping, are required for the development of mechanistic hypotheses. In the absence of such data, *HCAR1*, *ATG10*, *GALNT2*, *BICC1*, *EFNA5*, and *HIPK2* should be regarded as genomic candidates rather than as functionally validated determinants of body composition.

### 3.4. Autozygosity and Implications for Conservation Planning

The >30-fold range in FROH across Vietnamese breeds identifies Soc (14.46%), Co (11.58%), and Hung (9.75%) as the breeds warranting particular attention in conservation planning on the basis of elevated autozygosity, which may increase the risk of exposing deleterious recessive alleles and of inbreeding depression. It is important to note that FROH reflects autozygosity and inbreeding history only. Conservation priority cannot be established from FROH alone and requires additional information on effective and census population size, demographic history, genetic uniqueness, geographic distribution, cultural importance, and production value; the rankings presented here should therefore be read as one genomic input to conservation planning rather than as a priority list. The low FROH of Lung (~0.5%) and Meo (~1.1%) indicates these breeds maintain substantial genetic diversity, making them potential donors for diversity restoration programs. The contrast between Soc (high FROH, extreme PC2 position, highest within-Vietnamese FST vs. lean breeds) and Mong Cai (moderate FROH, distinct genomic profile at body size loci) underscores that within-Vietnamese differentiation reflects both local adaptation and demographic isolation, and that these two processes must be disentangled in conservation planning. Comprehensive conservation planning should additionally weigh breed census size, cultural and economic importance, geographic isolation, and phenotypic uniqueness alongside FROH, consistent with international guidelines for livestock genetic resource management.

The ROH length class analysis provides temporal resolution to the inbreeding signals detected by FROH. The predominance of long ROHs (>8 Mb) across most Vietnamese breeds indicates that elevated autozygosity reflects recent inbreeding, driven by small effective population sizes and geographic isolation, rather than ancient demographic bottlenecks [25,26,27]. This distinction is conservation-critical: recent inbreeding is more amenable to intervention through managed crossing programs than deep historical drift [25,26,27]. The extreme long ROH accumulation in Soc and Co, combined with their high FROH (Section 3.5), identifies these breeds as those in which unmanaged inbreeding may expose deleterious recessive alleles within a small number of generations and therefore as breeds warranting particular attention in conservation planning. Further studies on effective population size trajectories in Soc and Co are required for the development of evidence-based minimum viable population recommendations.

### 3.5. Haplotype-Level Description of Candidate Loci

Haplotype bifurcation analysis provided haplotype-level visualization of the FST and XP-EHH signal at the *GPC5* locus. Because the same phased genotypes underlie all three analyses, this visualization illustrates the signal descriptively and does not constitute independent validation. At *GPC5* (Chr11:61,553,605), Northern Vietnamese breeds exclusively carried the ancestral allele with a dense haplotype trunk extending across approximately 50 Mb, a haplotype pattern consistent with recent positive selection (Figure S2); the data do not allow a hard sweep, in which a single advantageous haplotype rises to fixation, to be distinguished from fixation by drift at *n* = 6. *GPC5* encodes glypican−5, a heparan sulfate proteoglycan that potentiates IGF and FGF signaling at the cell surface; selection at this locus may therefore reflect adaptation of growth axis signaling to the low-nutrient highland environments of Northern Vietnam. However, no nutritional, growth, or environmental phenotypes were measured in this study, and this interpretation therefore remains a hypothesis generated by the genomic signal rather than a demonstrated adaptive mechanism.

The *BNC2* (Basonuclin-2) locus (Chr1:206 Mb) showed elevated FST in the within-Vietnamese comparison but negative XP-EHH values in the Northern vs. Landrace analysis, indicating that the extended haplotype signal at this position was in the Landrace rather than the Vietnamese direction. *BNC2* encodes a zinc finger transcription factor with established roles in melanocyte regulation and skin pigmentation [28,29,30], and its elevated within-Vietnamese FST is consistent with the striking coat color diversity among Vietnamese breeds. However, given the absence of a positive XP-EHH signal in Vietnamese breeds, BNC2 should be considered a differentiation candidate rather than a selective sweep locus, and further functional studies on BNC2 expression in Vietnamese pig skin are required. In contrast, Landrace haplotypes at the *GPC5* locus showed immediate branching with no extended homozygosity, consistent with, but not establishing, the absence of recent selection at this locus in the six sampled Landrace individuals (Figure S2). The *WNT16*/*FAM3C*/*CPED1* region (Chr18:25.4–25.6 Mb) was identified by phenotypic XP-EHH as a signal distinguishing lean breeds from extreme fat/prolific breeds rather than from medium-bodied breeds (Section 2.6), and *WNT16* and *CPED1* have established roles in skeletal morphology and bone mineral density [31]. The fixation of a distinct haplotype at this region in lean breeds relative to extreme fat/prolific breeds is consistent with directional selection for compact body size, though this breed contrast differs from earlier reports and warrants further validation.

### 3.6. Functional Enrichment: Pathway-Level Patterns Among Candidate Genes

The GO and pathway enrichment results provide functional context for the candidate genes identified by FST and XP-EHH analyses, moving from individual gene-level signals to pathway-level biological narratives. The three Vietnamese vs. Landrace comparisons shared a common core of 41 enriched biological processes, including anatomical structure development, system development, multicellular organism development, and developmental process (FDR < 0.05), reflecting the deep evolutionary divergence between Asian and European pig lineages and consistent with selection on growth trajectories and body composition during domestication. The Northern vs. Landrace comparison additionally showed strong enrichment for regulation of response to stimulus (*p* = 1.95 × 10^−8^) and cell surface receptor signaling pathway (*p* = 1.51 × 10^−4^), consistent with selection on environmental sensing and stress response in Northern Vietnamese highland breeds. These broad developmental GO:BP terms indicate that selection has targeted the regulatory architecture of growth and differentiation, rather than specific metabolic enzymes, a pattern consistent with previous reports of selection on developmental gene networks during pig domestication. The GO enrichment analysis identified macrophage migration and myeloid leukocyte chemotaxis as the most specific biological processes enriched in the Northern vs. Southern comparison, consistent with the KEGG-level innate immune signal. These results are in agreement with the identification of *CXCL8*, *TLR4*, *RELA*, *CCR1*, and *CCR2* as core Northern vs. Southern candidate genes (Section 3.4), identifying immune-related genomic differentiation between Northern highland and Southern lowland breeds that is potentially consistent with differential pathogen-related selection. Because pathogen exposure, immune phenotypes, and environmental variables were not measured in this study, differential pathogen pressure is offered as a hypothesis rather than as an established driver of within-Vietnamese genomic divergence.

Furthermore, convergent enrichment of four innate immune KEGG pathways in the Northern vs. Southern comparison, all sharing *CXCL8*, *TLR4*, *RELA*, and *CCR1*/*CCR2* as core genes, identifies immune-related genomic differentiation potentially consistent with differential pathogen-related selection between these groups; in the absence of pathogen exposure or immune phenotype data, this cannot be established as an axis of within-Vietnamese breed differentiation. Chemokines orchestrate the recruitment of immune cells to sites of infection and inflammation [32,33]; selection on chemokine receptor and cytokine genes would be expected under differential pathogen pressure across Vietnam’s geographic zones, from the cool humid highlands of the north to the tropical lowlands of the south.

The Type II diabetes mellitus pathway enrichment in the Northern vs. Central comparison (BH-adjusted *p* = 0.0023), driven by co-located *ABCC8*/*KCNJ11* (pancreatic ATP-sensitive potassium channel) and *IRS1* (insulin receptor substrate 1), suggests divergent selection on glucose metabolism and insulin secretion between Northern highland foraging breeds and Central coastal agricultural breeds. This may reflect adaptation to contrasting dietary carbohydrate availability across Vietnam’s geographic zones [34]. Similar metabolic pathway divergence between highland and lowland livestock has been reported in yaks [35], where dietary energy and protein gradients altered carbohydrate, fat, and amino acid metabolism, and in Tibetan pigs, where highland-versus-lowland comparisons revealed differences in metabolic pathways, including carbohydrate metabolism [36]. These similarities suggest that diet-driven metabolic adaptation may be a conserved feature of geographic diversification in Asian livestock. However, it is not clear whether the T2D pathway enrichment reflects selection on insulin secretion specifically or on broader glucose homeostasis, and further studies examining metabolic phenotypes in Northern highland and Central coastal Vietnamese breeds are required for the development of mechanistic hypotheses. It is important to note that the small sample size of the Central group (*n* = 12 individuals) might have influenced the FST estimates at these loci, and replication in larger cohorts is recommended.

The Northern vs. Southern KEGG analysis reveals a unified innate immune defense signature. *CXCL8* (Chr8; FST = 0.445), *TLR4* (Chr1; FST = 0.336), RELA (Chr2; FST = 0.282), and *CCR1*/*CCR2* (Chr13; FST = 0.219) are core components of innate immune responses to bacterial pathogens [37,38,39,40] and collectively implicate pattern recognition, NF-κB transcriptional activation, and chemokine-driven immune cell recruitment as key axes of genomic differentiation between Northern highland and Southern lowland breeds. It is important to note that these KEGG pathways were identified by a supplementary clusterProfiler analysis using Benjamini–Hochberg correction rather than the g:SCS correction applied elsewhere; only pathways meeting FDR < 0.05 are reported. All four meet that criterion, but the differing correction methods mean they are not directly comparable with the g:Profiler results, and they should be considered exploratory and interpreted alongside the stronger GO:BP immune signals rather than as definitive pathway-level conclusions. Further studies on immune gene expression and pathogen resistance phenotypes in Northern highland and Southern lowland Vietnamese breeds are required for the development of mechanistic hypotheses.

### 3.7. Methodological Considerations and Limits of Interpretation

Perhaps the major limitation of this study is the small sample size of six individuals per breed, which constrains every statistic reported here rather than merely reducing power. With *n* = 6, allele frequencies are estimated from at most twelve chromosomes per locus, so per-SNP FST carries large sampling variance and can approach its theoretical maximum at markers that are differentially fixed by drift alone; XP-EHH and iHS are estimated from between twelve and 120 haplotypes depending on the grouping, which limits the resolution with which extended haplotype homozygosity can be distinguished from the haplotype structure expected with drift in a small sample, and FROH estimates carry wide within-breed dispersion, as reflected in the standard deviations reported in Table 4 (for example, Hung: FROH = 9.75%, SD = 10.13; Van_Pa: FROH = 6.14%, SD = 7.21). We addressed this in part by pooling breeds into regional and phenotypic groups, though this introduces assumptions of within-group homogeneity that the data cannot test. Therefore, the candidate loci, adaptive interpretations, and conservation inferences presented above are exploratory population-genomic signals requiring replication in larger cohorts. In addition, the use of Landrace as the sole European reference is a further constraint on interpretation. Six Landrace individuals drawn from a single commercial breed cannot represent the genetic diversity of European commercial pigs, and the differences reported between Vietnamese breeds and Landrace therefore reflect an unresolved combination of Asian–European divergence, breed-specific selection within the Vietnamese populations, Landrace-specific selection, and the demographic history of the sampled Landrace animals. Because additional European reference breeds were not available in this dataset, all statements concerning divergence from European commercial lineages should be read as divergence relative to the sampled Landrace population, and the same restriction applies to Ba Xuyen, whose suggested European genetic contribution is defined relative to this single reference. Further studies incorporating multiple European commercial and local breeds are required for the development of a general account of Asian–European differentiation in this system.

Moreover, it is important to distinguish explicitly between analytical robustness and biological validation. Threshold sensitivity analysis confirmed that *TLR4*, *GPC5*, *E2F6*, *NOS1*, *ABL1*, *BCAT1*, and *ABCA1* were recovered at both the 90th (102 windows) and 95th (20 windows) percentile thresholds, and the 99th percentile returned zero windows, consistent with expected behavior at *n* = 6 and medium-density SNP data. Within-population iHS, which detects unusually extended haplotypes surrounding high-frequency alleles within a single population [41], was elevated at *E2F6* (Northern-specific), *NOS1* (pan-Vietnamese), *HCAR1* (strongest in Northern and also above threshold in Central), and *GALNT2* (Northern and Southern). Nevertheless, all of these analyses were performed on the same 96 individuals and the same 48,896-SNP dataset.

The rationale for integrating FST and XP-EHH is that the two statistics are sensitive to different features of a sweep [42]: FST captures allele-frequency divergence between populations irrespective of haplotype context, whereas XP-EHH captures the extended haplotype homozygosity expected when an allele has risen in frequency rapidly in one population relative to another. Requiring a window to exceed the 95th percentile of both distributions is therefore a heuristic intersection filter that reduces the proportion of windows selected by drift alone; it is not a formal joint test, no combined null distribution was constructed, and no genome-wide significance level can be attached to the resulting windows. Furthermore, several properties of the data may affect the signals reported here. The PorcineSNP60v2 array ascertains common variants largely in European commercial breeds, so low-frequency and rare variants segregating in Vietnamese populations are under-represented and causal variants are unlikely to have been genotyped directly. The MAF > 0.05 filter applied before XP-EHH and iHS removed a further fraction of markers (33,588 and 27,837 SNPs retained in the Northern and Central comparisons, respectively), reducing power at loci where a selected haplotype segregates at low frequency. Uneven SNP density and local variation in recombination rate influence the length of observed homozygosity tracts independently of selection, and both demographic history and background selection can generate elevated FST and extended haplotypes in the absence of positive selection [43]. Nevertheless, the combination of statistics used here strengthens the descriptive evidence for differentiation at these loci; it does not establish that they are true selective sweeps.

The phenotypic component of this study is a breed-level comparison. Individual measurements of body weight, backfat thickness, fat percentage, carcass composition, and reproductive performance were not available for these animals, and breeds were assigned to the lean/small-bodied, extreme-fat/prolific, and medium-bodied groups on the basis of published breed descriptions and coat color. The XP-EHH contrasts therefore quantify genomic differentiation among breeds classified into phenotype-related groups, and they cannot establish association between genotype and individual body-composition phenotypes. It is important to note that coat color, body size, fat deposition, and geographic origin are correlated among Vietnamese breeds, so the phenotypic and regional contrasts are not statistically independent, and a signal attributed here to a body-composition contrast might instead reflect regional ancestry. Further studies combining individual phenotypic records with genome-wide genotypes are required for the development of genotype–phenotype associations in these breeds.

Finally, no functional evidence is presented for any candidate gene. The functions attributed to *HCAR1*, *ATG10*, *GPC5*, *GALNT2*, *E2F6*, *NOS1*, *TLR4*, and the remaining candidates were taken from public annotation and from studies in other species and tissues, and the mechanistic accounts discussed above are hypotheses following from those annotations rather than findings of the present study. All of these genes are therefore genomic candidates, not functionally validated genes. Experimental validation, including qPCR or RNA-sequencing quantification of candidate gene expression in relevant tissues, allele-specific expression analysis, and, where feasible, functional perturbation, is required before any of these genes can be described as functionally involved in the traits discussed here. Larger cohorts, whole-genome sequencing, individual phenotypic data, formal admixture analysis, and multiple European reference populations are likewise required before firm conclusions can be drawn.

## 4. Materials and Methods

### 4.1. Data and Animal Classification

The population information and methods to collect data have been described in [14]. Genotype data were obtained from a publicly available dataset comprising 96 animals: 90 pigs from 15 local Vietnamese breeds sampled across 15 geographically distinct locations and 6 Landrace pigs from Hanoi as a European commercial outgroup [14]. All animals were genotyped using the Illumina PorcineSNP60v2 BeadChip (San Diego, CA, USA) (~62,000 SNPs, Sus scrofa Sscrofa11.1 assembly) [44]. A sample naming inconsistency (one Landrace animal labeled Land_v5 vs. Land_V for others) was standardized prior to analysis. For regional analyses, Vietnamese breeds were classified into three geographic groups based on verified provincial origins: Northern (10 breeds, *n* = 60: Meo, Hung, Muong_Khuong, Ban, Lung_Pu, Ha_Lang, Mong_Cai, Lung, Tap_Na, Huong), Central (2 breeds, *n* = 12: Van_Pa, Co), and Southern (3 breeds, *n* = 18: Ba_Xuyen, Soc, Chu_prong). Landrace (*n* = 6) served as the European reference. Because Landrace (*n* = 6) and the Central group (*n* = 12) are small relative to Northern (*n* = 60), comparisons involving fewer than 20 individuals should be interpreted as exploratory rather than conclusive. It is important to note that Landrace is a single European commercial breed and cannot represent the genetic diversity of European commercial pigs; no additional European reference population was available in this dataset. Differentiation reported between Vietnamese breeds and Landrace may therefore reflect a combination of Asian–European divergence, breed-specific selection in the Vietnamese populations, Landrace-specific selection, and demographic history, and all conclusions regarding divergence from European commercial lineages are restricted to differentiation relative to the sampled Landrace population.

### 4.2. Quality Control

Raw genotype data were processed with PLINK v1.90b7 [45] applying autosome extraction (chromosomes 1–18); SNP missingness --geno 0.10 then 0.05; individual missingness --mind 0.10; Hardy–Weinberg equilibrium *p* < 1 × 10^−6^; MAF > 0.01. The individual-level genotyping rate and observed heterozygosity were computed using PLINK --missing and --het (Supplementary Table S1). No individual exceeded 10% missingness and no heterozygosity outlier was detected. Relatedness was assessed using pi-hat (threshold 0.25; no related pairs identified), and no duplicate individuals were found (Table S2). The per-breed mean genotyping rate ranged from 0.992 (Soc) to 0.998 (Landrace), and mean observed heterozygosity ranged from 0.164 (Soc) to 0.388 (Ba_Xuyen), the latter markedly higher than all local Vietnamese breeds, providing suggestive evidence of a European genetic contribution. QC in this study assessed relatedness, heterozygosity, and missingness; sample contamination and sex-chromosome concordance were not separately screened as the analysis was restricted to autosomal SNPs (chromosomes 1–18) throughout.

### 4.3. Principal Component Analysis

SNPs were LD-pruned (window 50, step 10, r2 < 0.1), yielding 9901 SNPs, and PCA was performed on this pruned set using PLINK --pca (20 components) [45]. All 48,896 post-QC SNPs were retained for FST, XP-EHH, and ROH analyses. Breed centroids were computed as the mean PC score across individuals within each breed.

### 4.4. Regional Pairwise FST

Pairwise FST was estimated using the Weir and Cockerham (1984) estimator [46] in PLINK (v1.90b7) for six regional comparisons. Per-SNP FST values were computed (negative values set to zero), and windowed FST was computed in 100 kb non-overlapping windows (minimum 3 SNPs) using weighted means. Outlier windows exceeded the 99th percentile.

### 4.5. Haplotype Phasing and XP-EHH

Duplicate variants were removed, and data were phased chromosome-by-chromosome using BEAGLE [47]. XP-EHH was computed using the rehh R package (v3.2.2) [48] for Northern vs. Landrace and Central vs. Landrace comparisons (MAF > 5%, 33,588 and 27,837 SNPs, respectively). High-confidence windows exceeded the 95th percentile in both windowed FST and XP-EHH for the same comparison. This intersection was applied as a heuristic filter intended to reduce the proportion of windows selected by drift alone and not as a formal joint test; no combined null distribution was constructed, and no genome-wide significance level is attached to these windows.

### 4.6. Within-Vietnamese FST Analysis

To identify genomic signatures of within-Vietnamese phenotypic diversification, breeds were classified based on published phenotypic descriptions (Nguyen et al., 2026) [2] and coat color phenotype into three groups: lean/small-bodied (*n* = 42: Ban, Hung, Lung, Van_Pa, Co, Soc, Chu_prong; predominantly black-coated), extreme fat/prolific (*n* = 18: Mong_Cai, Huong, Ha_Lang; white or light-coated, large-bodied, high fat deposition), and medium-bodied (*n* = 24: Muong_Khuong, Lung_Pu, Tap_Na, Meo). XP-EHH was computed for four pairwise comparisons: lean vs. extreme fat/prolific, lean vs. medium-bodied, lean vs. all prolific combined (*n* = 42), and medium vs. extreme fat/prolific. The medium vs. extreme comparison was included to distinguish signals specific to the extreme fat phenotype from those shared across all large-bodied breeds. Mong_Cai was additionally analyzed individually against the lean group. Population-specific phased VCFs were generated for each group using PLINK v1.9 from BEAGLE-phased genotypes, and XP-EHH was computed using the rehh package v3.2.2 in R with a minimum MAF of 0.05 and 100 kb sliding windows. Meo was assigned to the medium-bodied group on the basis of its published body size (boars 80–120 kg, sows 70–100 kg), which is intermediate between the lean/small-bodied breeds and the extreme fat/prolific breeds. Ba Xuyen was not assigned to a phenotypic group and was excluded from this analysis because its markedly elevated heterozygosity and its intermediate position in principal component space indicate a European genetic contribution (Section 2.1 and Section 2.2) that would confound a phenotype-based haplotype contrast with ancestry. The phenotypic comparisons therefore comprise 84 of the 90 Vietnamese animals.

### 4.7. Gene Annotation

Candidate windows were annotated using the Sus scrofa Ensembl release 112 gene annotation (Sscrofa11.1 GTF). Overlaps with protein-coding gene coordinates were identified using the GenomicRanges R package [49].

### 4.8. Runs of Homozygosity

ROHs were detected using PLINK --homozyg (v1.90b7) [45] (minimum window 50 SNPs, max 1 heterozygous call per window, minimum ROHs 1 Mb, minimum 100 SNPs, maximum gap 1 Mb, minimum density 1 SNP/50 kb). FROH was calculated as total ROH length divided by the genome length.

### 4.9. Haplotype Bifurcation Analysis

To further characterize at the haplotype level, the candidate selection signatures identified by windowed FST and XP-EHH, and to visualize extended haplotype homozygosity at priority loci, a haplotype bifurcation diagram was constructed for GPC5 (Chr11:61,553,605), the highest-confidence Northern vs. Landrace candidate gene, using the calc_furcation() function in rehh v3.2.2. The diagram compared Northern Vietnamese breeds (*n* = 60 individuals, 120 haplotypes) against Landrace (*n* = 6 individuals, 12 haplotypes). Extended haplotype homozygosity was assessed qualitatively by the extent of unbranched parallel haplotype arms extending from the focal SNP in both directions; a candidate sweep pattern was defined as a dense, minimally branching haplotype trunk extending across more than 20 Mb on at least one allele.

### 4.10. Gene Ontology and Pathway Enrichment Analysis

To characterize the functional landscape of the selected genomic regions, we performed Gene Ontology (GO) and pathway enrichment analysis on candidate genes identified within the top 5% windowed FST windows for each pairwise regional comparison. Protein-coding genes overlapping these windows were extracted using the GenomicRanges R package [49] with Sus scrofa Ensembl release 112 annotation (Sscrofa11.1), yielding between 466 and 534 unique candidate genes per comparison. Enrichment analysis was performed using the gprofiler2 R package (v0.2.3) [17], which queries the g:Profiler web server with continuously updated Ensembl-based Sus scrofa (sscrofa) annotation. GO terms across all three ontologies (Biological Process, GO:BP; Molecular Function, GO:MF; Cellular Component, GO:CC), KEGG pathways, and Reactome (REAC) pathways were tested simultaneously for each pairwise comparison using the g:SCS multiple testing correction method (FDR-adjusted *p* < 0.05). Supplementary KEGG analysis was additionally performed using the clusterProfiler package (v4.8) [50] with the enrichKEGG function (organism code ssc; BH-adjusted *p* < 0.05). The g:Profiler approach was selected over the locally installed org.Ss.eg.db Bioconductor annotation package (v2.4.6) [51], which returned zero significant GO:BP terms during preliminary analysis, reflecting the historically incomplete functional annotation of the pig genome in static databases. The clusterProfiler KEGG analysis was applied to all six pairwise comparisons; the Northern vs. Southern contrast yielded the most biologically specific pathway signals, and Central vs. Southern, Central vs. Landrace and Southern vs. Landrace also returned significant enrichment with the corrected panel (Table 6). KEGG Entrez IDs were converted to gene symbols using the bitr function in clusterProfiler. Enrichment results were visualized as a combined GO:BP dot plot across all comparisons and as horizontal bar plots per comparison using ggplot2 (v3.4) in R (v4.5.2) [52].

### 4.11. iHS Analysis, f3-Statistics, and Threshold Sensitivity

Within-population iHS was computed for Northern, Central, and Southern groups using ihh2ihs() in rehh v3.2.2 (freqbin = 0.025, MAF > 0.05) [48]. |iHS| > 2 was used as the candidate sweep threshold. Windowed mean |iHS| was computed in 100 kb non-overlapping windows. To formally test Ba Xuyen admixture, f3(Ba_Xuyen; Landrace, Vietnamese_reference) was computed as the mean over SNPs of (p_BaXuyen–p_Landrace)(p_BaXuyen–p_Vietnamese), with Lung and Meo as the Vietnamese reference (lowest FROH). Allele frequencies were estimated using PLINK --freq. Standard error was estimated by block jackknife over 18 autosomal chromosomes, and a Z-score was computed as f3/SE. For the admixture f3 test, formal evidence of admixture requires a significantly negative f3 statistic; the criterion applied here was therefore f3 < 0 together with Z < −3. A positive f3 statistic does not provide evidence of admixture irrespective of the magnitude of its Z-score, and a large positive Z-score therefore cannot satisfy this criterion. Threshold sensitivity was assessed by repeating the high-confidence window criterion at the 90th and 99th percentile for Northern vs. Landrace. Threshold sensitivity results are provided in Supplementary Table S2; f3 admixture results in Supplementary Table S3; and iHS values at key candidate loci in Supplementary Table S4. Because iHS, XP-EHH, and FST were computed from the same individuals and the same post-QC SNP set, a locus is described as corroborated when it exceeded the relevant threshold in more than one of these statistics; such corroboration denotes analytical consistency within a single dataset and is not equivalent to independent biological replication.

## 5. Conclusions

In this study, we provide the first genome-wide characterization of genomic differentiation, candidate selection signatures, and autozygosity in local Vietnamese pig breeds. The conclusion most strongly supported by these data is that local Vietnamese breeds are genomically differentiated from one another and from the sampled Landrace population, and that this differentiation is concentrated in a defined set of genomic windows. Integration of FST and XP-EHH identified *GPC5*, *E2F6*, *NOS1*, *ABL1*, and *TLR4* as Northern candidate loci and *CRYM*/*ZP2* as the leading Central candidate locus; these windows were recovered at both the 90th and 95th percentile thresholds, and iHS computed on the same dataset was elevated at *E2F6* (Northern |iHS| = 3.04) and *NOS1* (pan-Vietnamese |iHS| = 2.66–3.36), which indicates analytical robustness rather than independent replication. Breed-level phenotypic XP-EHH identified *GALNT2* as a candidate shared across breed groups, *HCAR1* and *ATG10* as candidates specific to the extreme-fat/prolific breed group, and EFNA5 and HIPK2 as candidates specific to the medium-bodied breed group. Ba Xuyen showed elevated heterozygosity and an intermediate position in principal component space, providing suggestive evidence of a European genetic contribution; the f3 statistic was positive and therefore did not meet the formal criterion for admixture, which requires a significantly negative f3. ROHs identified Soc, Co, and Hung as the breeds with the highest autozygosity and therefore as breeds warranting particular attention in conservation planning. However, tropical adaptation, body-composition mechanisms, European admixture in Ba Xuyen, and conservation priority are presented here as interpretations requiring further validation rather than as demonstrated findings, because environmental and physiological phenotypes were not measured, individual body-composition phenotypes were not available, the f3 result did not support formal admixture, no candidate gene was functionally validated, and a single European breed served as the reference. All findings reported here therefore require replication in larger cohorts with whole-genome sequencing, individual phenotypic measurements, multiple European reference populations, formal admixture analysis, and functional validation.

## Figures and Tables

**Table 1:** Summary of QC filtering steps applied to the Illumina PorcineSNP60v2 genotype data.

Filter Step	Parameter	SNPs Removed	SNPs Remaining
Initial dataset	Raw SNP60v2		60,095
Autosome extraction	Chr 1–18 only	2586	57,509
Individual missingness	--mind 0.10	0	57,509
SNP missingness (pass 1)	--geno 0.10	3123	54,386
SNP missingness (pass 2)	--geno 0.05	1570	52,816
Hardy–Weinberg equilibrium	--hwe 1 × 10^−6^	908	51,908
Minor allele frequency	--maf 0.01	3012	48,896
Final dataset	After all filters		48,896

**Table 5:** Candidate genes in top within-Vietnamese phenotypic XP-EHH selection windows.

Gene(s)	Chr	Position (Mb)	XP-EHH * (Comparison)	Candidate Class
*GALNT2*	14	60.05	LvE = 2.50; LvM = 4.93; LvP = 4.52; MvE = 0.48 (below)	Shared across breed groups
*BICC1*	14	61.96	LvE = 4.21; LvM = 3.34; LvP = 4.03; MvE = 1.72 (at threshold)	Shared across breed groups **
*HCAR1*	14	30.11	LvE = 3.79; LvM = 1.50 (below); LvP = 1.88; MvE = 2.60	Extreme-fat/prolific group
*ATG10*	2	90.55	LvE = 4.42; LvM = 3.89; LvP = 4.95; MvE = 1.94	Extreme-fat/prolific group
*EFNA5*	2	112.68	LvE = 0.70 (below); LvM = 2.00; LvP = 1.25 (below); MvE = −0.14 (below)	Medium-bodied group
*HIPK2*	18	10.00	LvE = 1.44 (below); LvM = 3.25; LvP = 4.55; MvE = −0.20 (below)	Medium-bodied group
*WNT16*/*FAM3C*/*CPED1* region	18	25.50	LvE = 1.84; LvM = 1.33 (below); LvP = 2.21; MvE = 1.20 (below)	Comparison-specific (see values)

* LvE = lean vs. extreme (Mong_Cai, Huong, Ha_Lang; *n* = 18); LvM = lean vs. medium (Muong_Khuong, Lung_Pu, Tap_Na, Meo; *n* = 24); LvP = lean vs. prolific (the combined extreme-fat/prolific and medium-bodied groups, 18 + 24 = 42 animals, which coincidentally equals the size of the lean group); MvE = medium vs. extreme; MC = lean vs. Mong_Cai. Candidate class: shared across breed groups = signal in LvE and LvM but not MvE; extreme-fat/prolific group = signal in LvE and MvE; medium-bodied group = signal in LvM only. Ha_Lang classified with extreme group based on white coat color and phenotypic similarity to Huong (Nguyen et al., 2026) [2]. Windows in top 5% of 2+ comparisons are prioritized. Candidate classes are defined solely by the profile of XP-EHH signals across breed-level phenotypic groups and do not imply association with individual-level body-composition measurements, which were not available in this dataset. ** The medium vs. extreme value for *BICC1* (1.72) coincides with the 95th percentile threshold for that comparison (1.717). Its assignment between the shared and the extreme fat/prolific classes is therefore not resolved by these data, and it is retained in the shared class based on its lean vs. extreme, lean vs. medium and lean vs. prolific signals. Six further loci clearing the threshold in two or more comparisons (*SSBP2*, *ACOT12*, *LAMA3*, *ANKRD29*, *STXBP6*, *ABLIM3*), together with WNT16 evaluated as a gene body rather than as a region, are reported in Supplementary Table S10. *SSBP2* and *ACOT12* fall within the *ATG10* region and are therefore not independent signals.

**Table 6:** KEGG and Reactome pathway enrichment results from top 5% windowed FST candidate genes.

Comparison	Tool	Pathway	Genes (*n*)	Adj. *p*-Value	Top Gene (FST)
N vs. C	g:Profiler KEGG	Type II diabetes mellitus	8	3.45 × 10^−3^	*HK1* (0.266)
N vs. S	g:Profiler REAC	CDC42 GTPase cycle	12	0.030	*ARHGAP22* (0.332)
N vs. S	g:Profiler REAC	HS-GAG degradation	5	0.033	*GPC6* (0.340)
N vs. S	clusterProfiler *	Viral cytokine/receptor interaction	8	0.036	*CXCL8* (0.445)
N vs. S	clusterProfiler *	Pertussis	7	0.038	*CXCL8* (0.445)
N vs. S	clusterProfiler *	PI3K-Akt signaling pathway	16	0.038	*EREG* (0.474)
N vs. S	clusterProfiler *	Cadherin signaling pathway	17	0.038	*MAPK10* (0.352)
S vs. L	clusterProfiler *	African trypanosomiasis	6	0.043	*LAMA4* (0.524)
S vs. L	g:Profiler REAC	Neurotransmitter receptors and postsynaptic signal transmission	12	3.68 × 10^−2^	*NSF* (0.617)
S vs. L	g:Profiler REAC	Transmission across Chemical Synapses	15	3.68 × 10^−2^	*NSF* (0.617)
C vs. S	clusterProfiler *	Viral protein interaction with cytokine and cytokine receptor	9	0.036	*CSF1R* (0.313)
N vs. C	clusterProfiler *	Type II diabetes mellitus	8	0.0023	*HK1* (0.266)

* BH = Benjamini–Hochberg correction applied in clusterProfiler enrichKEGG. Top gene FST values are windowed FST (100 kb windows) from the relevant comparison. N = Northern, C = Central, S = Southern, L = Landrace. Breed assignment: Van_Pa and Co = Central; Chu_prong and Soc = Southern; Tap_Na and Huong = Northern. Full pathway gene lists are provided in Supplementary Table S7.

## Data Availability

Genotype data are publicly available from https://www.animalgenome.org/repository/pub/NIASJP2017.0920/ (Accessed on 20 November 2025).

## Supplementary Materials

The following supporting information can be downloaded at: https://www.mdpi.com/article/10.3390/ijms27177897/s1.
