# Supplementary material for: Genome-Wide Differentiation, Inbreeding, and Candidate Selection Loci in Local Vietnamese Pig Breeds
Source: Int J Mol Sci. 2026 Sep 4;27(17):7897. doi: 10.3390/ijms27177897 (PMC13566751; doi:10.3390/ijms27177897)
Supplement: Supplementary file 1 [file ijms-27-07897-s001.zip › ijms-4387101-supplementary.pdf]

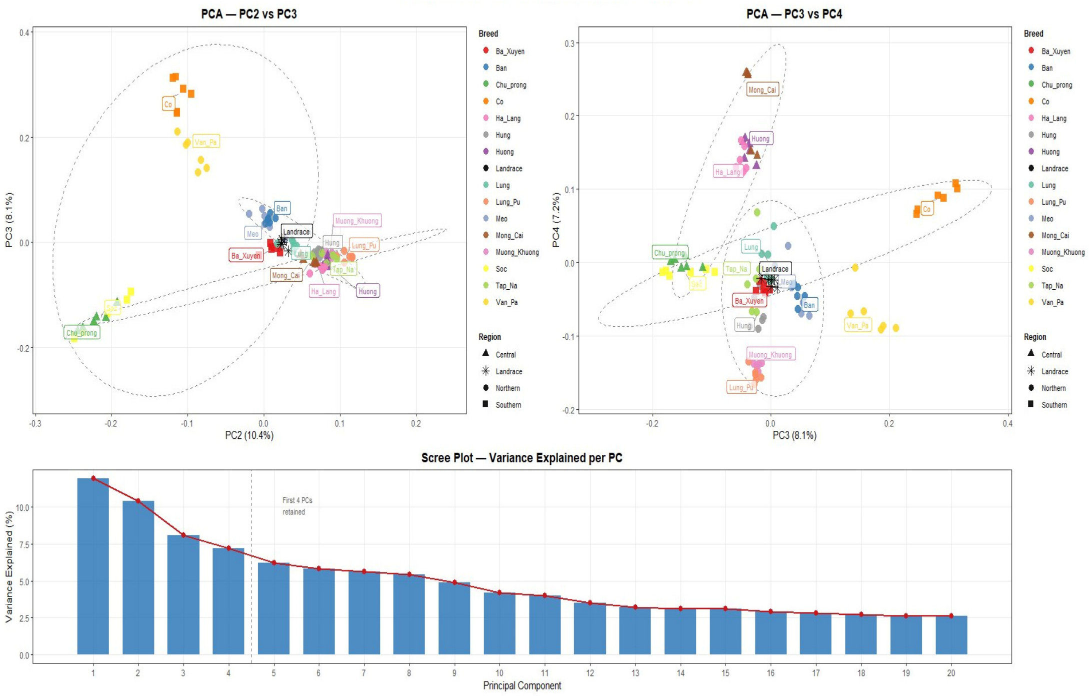

Figure S1. Extended principal component analysis of 15 Vietnamese local pig breeds and Landrace (n = 96 individuals, 9,901 LD-pruned SNPs post-QC). (A) PC2 vs PC3 (10.4% and 8.1% variance explained) reveals within-Vietnamese geographic structure independent of the Asian-European divergence captured by PC1, with Co and Van_Pa occupying distinct positions on PC3 and Chu_prong separating clearly along PC2. (B) PC3 vs PC4 (8.1% and 7.2%) shows fine-scale breed-level differentiation, with Mong_Cai clearly separating from Northern breeds along PC4, consistent with the within-Vietnamese FST signals at body size loci (Section 3.6). (C) Scree plot of variance explained per principal component; PC1 and PC2 explain similar proportions of variance (11.9% and 10.4% respectively), indicating multiple co-equal axes of genomic differentiation. Dashed ellipses represent 80% confidence regions per geographic group.

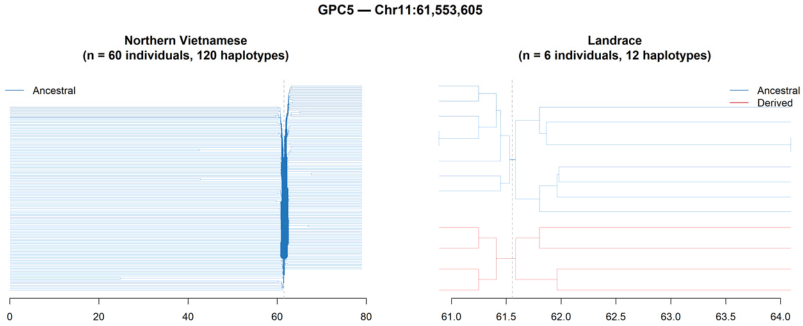

Figure S2. Haplotype bifurcation diagram at the GPC5 locus (Chr11:61,553,605) comparing Northern Vietnamese breeds (n = 60 individuals, 120 haplotypes; left panel) and Landrace (n = 6 individuals, 12 haplotypes; right panel). Each

horizontal line represents one phased haplotype. Dashed vertical line = focal SNP. Blue = ancestral allele. Northern Vietnamese breeds show a dense, unbranched single haplotype trunk consistent with extended haplotype homozygosity and with a candidate selective-sweep signal, while Landrace haplotypes branch immediately; given the small Landrace sample (n = 6), the latter pattern is consistent with, but does not establish, the absence of a selection signal at this locus.

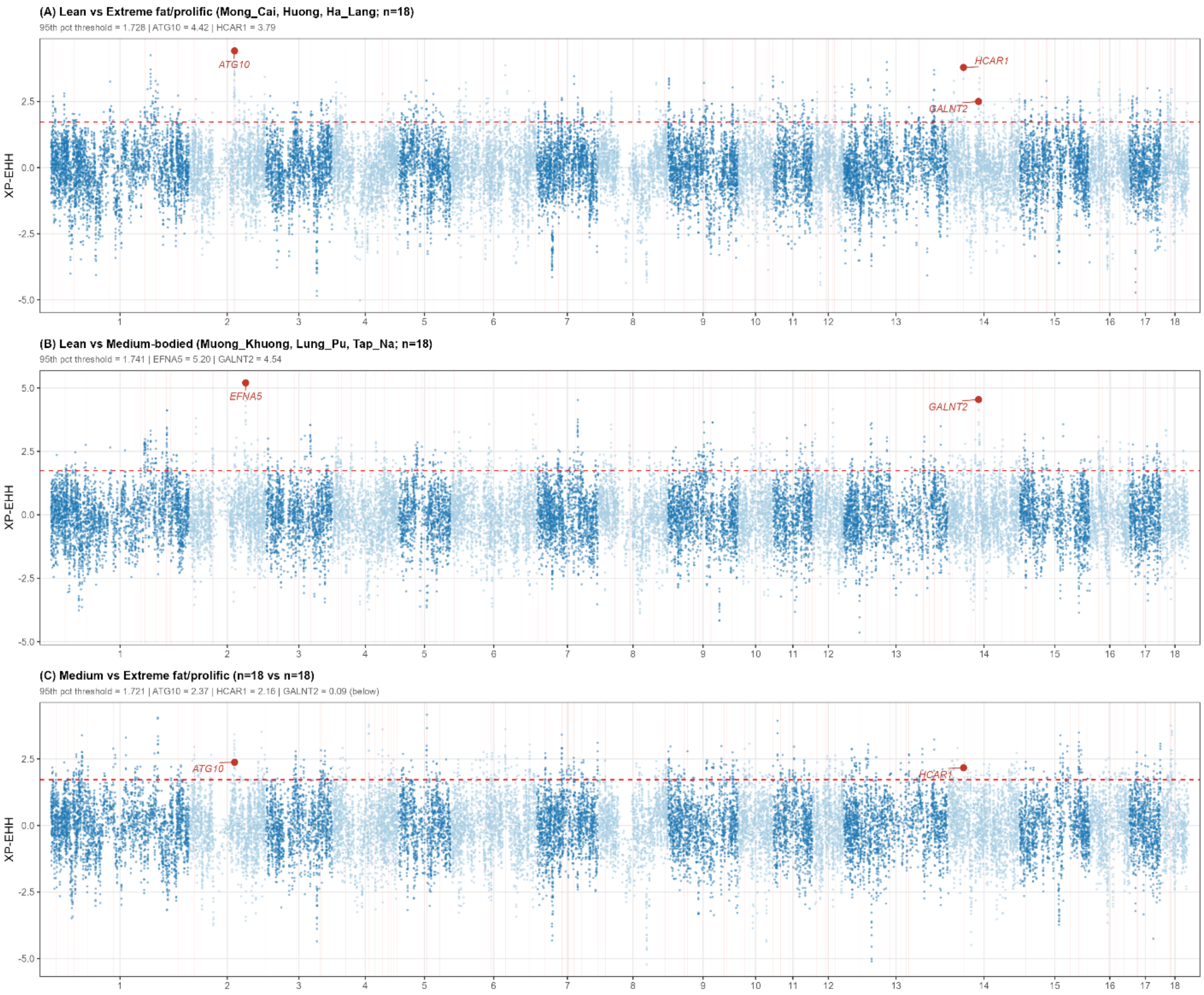

Figure S3. XP-EHH Manhattan plots for within-Vietnamese phenotypic comparisons. (A) Lean vs Extreme fat/prolific (n=42 vs n=18: Mong_Cai, Huong, Ha_Lang). (B) Lean vs Medium-bodied (n=42 vs n=24: Muong Khuong, Lung Pu, Tap Na, Meo). (C) Medium vs Extreme fat/prolific (n=24 vs n=18). Red dashed line = 95th percentile threshold (A = 1.728, B = 1.765, C = 1.717); red shading = top 5% windows. Panel C identifies HCAR1 (Chr14:30.1 Mb; XP-EHH = 2.60) and ATG10 (Chr2:90.55 Mb; XP-EHH = 1.94) as loci specific to the extreme-fat/prolific breed group: both exceed the threshold in panel C, indicating extreme fat/prolific breeds carry distinct haplotypes relative to medium-bodied breeds. ATG10 also exceeds the threshold in panel B (3.89), whereas HCAR1 does not (1.50); it is their panel C signal that distinguishes both from candidates shared across breed groups. GALNT2 (Chr14:60.05 Mb) exceeds the threshold in panels A (2.50) and B (4.93) but not panel C (0.48),

consistent with its classification as a candidate shared across breed groups. EFNA5 (Chr2:112.68 Mb) exceeds the threshold in panel B only, consistent with specificity to the medium-bodied breed group. All groups are breed-level phenotypic categories rather than individual body-composition measurements.

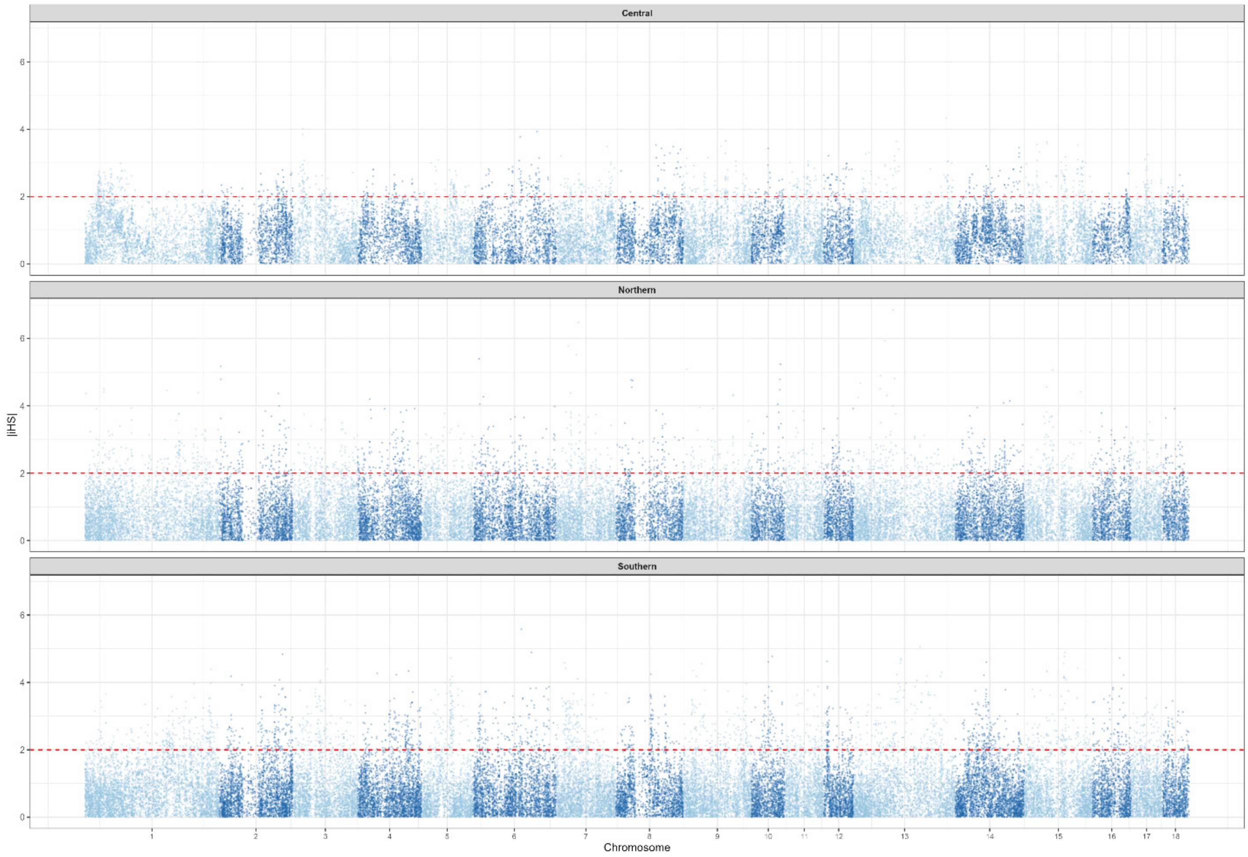

Figure S4. Within-population integrated haplotype score (iHS) Manhattan plots for the three Vietnamese regional groups. Each panel shows |iHS| values for all autosomal SNPs across chromosomes 1–18 for (A) Central, (B) Northern, and (C) Southern Vietnamese breeds. The red dashed line indicates the conventional |iHS| = 2 candidate sweep threshold. Key candidate loci identified by cross-population XP-EHH analysis were supported by an additional statistic within the same dataset, showing iHS signals above threshold; because iHS and XP-EHH were computed from the same individuals and the same genotypes, this indicates cross-method consistency rather than independent biological replication: E2F6 (Chr3:125.1 Mb) shows a Northern-specific signal (|iHS| = 3.04) absent in Central and Southern groups; NOS1 (Chr14:35.3 Mb) shows strong signals across all three groups (Northern |iHS| = 3.18, Central |iHS| = 2.66, Southern |iHS| = 3.36), consistent with pan-Vietnamese selection; HCAR1 (Chr14:30.1 Mb) shows the strongest Northern signal (|iHS| = 3.70), a weaker Central signal (|iHS| = 2.56) and no above-threshold Southern signal (|iHS| = 1.58); GALNT2 (Chr14:60.0 Mb) shows signals in Northern (|iHS| = 3.01) and Southern (|iHS| = 2.48) breeds, consistent with its classification as a candidate shared across breed groups. iHS was computed using the ihh2ihs() function in rehh v3.2.2 with freqbin = 0.025 and minimum MAF = 0.05.
